# B7-H3–Targeting Chimeric Antigen Receptors Epstein-Barr Virus–specific T Cells Provides a Tumor Agnostic Off-The-Shelf Therapy Against B7-H3–positive Solid Tumors

**DOI:** 10.1158/2767-9764.CRC-23-0538

**Published:** 2024-06-04

**Authors:** Siok Ping Yeo, Lindsay Kua, Jin Wei Tan, Joanna Kristyn Lim, Fiona HS Wong, May Delos Santos, Chek Meng Poh, Angeline XH Goh, Xin Yu Koh, Xiaohua Zhou, Ravisankar Rajarethinam, Qingfeng Chen, Zhisheng Her, Ivan D. Horak, Lionel Low, Kar Wai Tan

**Affiliations:** 1 Tessa Therapeutics Ltd, Singapore.; 2 Tikva Allocell Pte Ltd, Singapore.; 3 Institute of Molecular and Cell Biology (IMCB), Agency for Science, Technology and Research (A*STAR), Singapore, Republic of Singapore.

## Abstract

Encouraged by the observations of significant B7-H3 protein overexpression in many human solid tumors compared to healthy tissues, we directed our focus towards targeting B7-H3 using chimeric antigen receptor (CAR) T cells. We utilized a nanobody as the B7-H3–targeting domain in our CAR construct to circumvent the stability issues associated with single-chain variable fragment–based domains. In efforts to expand patient access to CAR T-cell therapy, we engineered our nanobody-based CAR into human Epstein-Barr virus–specific T cells (EBVST), offering a readily available off-the-shelf treatment. B7H3.CAR-armored EBVSTs demonstrated potent *in vitro* and *in vivo* activities against multiple B7-H3–positive human tumor cell lines and patient-derived xenograft models. Murine T cells expressing a murine equivalent of our B7H3.CAR exhibited no life-threatening toxicities in immunocompetent mice bearing syngeneic tumors. Further *in vitro* evaluation revealed that while human T, B, and natural killer cells were unaffected by B7H3.CAR EBVSTs, monocytes were targeted because of upregulation of B7-H3. Such targeting of myeloid cells, which are key mediators of cytokine release syndrome (CRS), contributed to a low incidence of CRS in humanized mice after B7H3.CAR EBVST treatment. Notably, we showed that B7H3.CAR EBVSTs can target B7-H3–expressing myeloid-derived suppressor cells (MDSC), thereby mitigating MDSC-driven immune suppression. In summary, our data demonstrate that our nanobody-based B7H3.CAR EBVSTs are effective as an off-the-shelf therapy for B7-H3–positive solid tumors. These cells also offer an avenue to modulate the immunosuppressive tumor microenvironment, highlighting their promising clinical potential in targeting solid tumors.

**Significance:**

Clinical application of EBVSTs armored with B7-H3–targeting CARs offer an attractive solution to translate off-the-shelf CAR T cells as therapy for solid tumors.

## Introduction

The emergence of chimeric antigen receptor (CAR) T-cell therapies targeting CD19 and B-cell maturation antigen (BCMA) signify a clinical breakthrough for the treatment of hematologic malignancies (1). However, translating CAR T-cell therapy into treatments for patients with solid tumors has been less successful. Among challenges that include heterogenous expression of tumor-associated antigens (TAA), CAR T-cell trafficking, and immunosuppressive tumor microenvironments (TME), one of the biggest obstacles remains the identification of appropriate TAAs in solid tumors. As most TAA can be found on nonmalignant tissues, albeit at relatively low levels, targeting such antigens may be associated with on-target, off-tumor toxicities (2). Therefore, selecting the appropriate TAA is a critical consideration to ensure safety and clinical efficacy of CAR T-cell therapy in solid tumors.

The B7 homolog 3 protein (B7-H3, CD276) is an immune checkpoint member of the B7 and CD28 families. In humans, B7-H3 exists in two isoforms: the shorter 2-Ig isoform comprises a single pair of immunoglobulin variable (IgV)-like and immunoglobulin constant (IgC)-like domains, while the dominant 4-Ig isoform consists of two identical IgV-IgC pairs due to exon duplication (3). In mice, B7-H3 exists as a single pair of IgV-IgC and shares 87% sequence homology with the human 2-Ig isoform (4). While limited protein expression of B7-H3 is detected in normal tissues, B7-H3 is overexpressed in multiple types of human tumors, tumor-infiltrating blood vessels, and tumor stroma (3). A strong correlation between high expression of B7-H3 protein and poor prognosis have been observed (5), supported by mechanistic studies that demonstrated a pleiotropic pro-oncogenic role for B7-H3 that is independent of its immune modulatory function (6). As a result of these observations, targeting B7-H3 has emerged as an appealing strategy for cancer immunotherapy. In addition to approaches that utilize conventional and newer generations of therapeutic antibodies to target B7-H3, some groups have also embarked on the development of B7-H3–targeting CAR T cells (7).

Antigen recognition by CAR T cells is mediated via an antigen-specific domain, commonly a single-chain variable fragment (scFv) of an antibody fused to transmembrane and intracellular signaling domains (8). It has been recognized in recent years that scFv aggregation arising from structural instability (9, 10) and domain swapping (11) can induce tonic signaling, which in turn diminishes CAR T activity and persistence. In comparison to traditional scFv, nanobodies derived from the variable domain of heavy chain-only antibodies (VHH) have more favorable stability qualities, while maintaining a high affinity to antigens. Using nanobodies in CAR constructs could potentially overcome the drawbacks associated with scFv-based targeting domains (12, 13).

The CD19 and BCMA-targeted CAR T-cell products currently approved by the FDA are generated from patient-derived T cells, which pose several challenges that limit their broader applications. Autologous CAR T-cell therapies are encumbered by bespoke manufacturing processes, unpredictable potency, risks of manufacturing failure, and a lengthy vein-to-vein time. Allogeneic CAR T cells derived from healthy donors may circumvent such hurdles as they are readily available as an “off-the-shelf” CAR T product with more consistent cell quality (14, 15). Recent studies of off-the-shelf allogeneic CD19-, CD7-, and BCMA-targeted CAR T therapy have reported encouraging clinical responses and a good safety profile in heavily pretreated patients (16–18).

To avoid GVHD, many allogeneic CAR T-cell products rely heavily on gene editing tools to eliminate endogenous αβ T-cell receptor (TCR), which inherently carries inadvertent risks of chromosomal alterations (19, 20). To avoid the use of genomic editing, we used T-cell products that naturally lack alloreactivity. Encouraged by the promising efficacy and safety outcomes of off-the-shelf Epstein-Barr virus–specific T cells (EBVST) in clinical trials (21) and the recent FDA approval of tabelecleucel, we adopted EBVSTs as universal donor T cells and armored them with a new-generation nanobody-based CAR targeting B7-H3. Our study demonstrates the promising *in vitro* and *in vivo* efficacy of our B7H3.CAR EBVST in targeting various human solid tumor types that express B7-H3. We further demonstrate that in addition to an acceptable on-target off-tumor safety profile, our candidate carries a low risk of treatment-induced cytokine release syndrome (CRS). Finally, our study reveals a novel function of B7H3.CAR EBVST, specifically its ability to target and reverse the immunosuppressive effects of myeloid-derived suppressor cells (MDSC).

## Materials and Methods

### B7-H3 Staining on Healthy Tissue and Tumor Microarrays

Formalin-fixed paraffin-embedded (FFPE) human healthy tissues (FDA331, TissueArray), gastric cancer, and triple-negative breast cancer (TNBC) microarrays with matched healthy tissues (Singhealth Tissue Repository) were sent to Advanced Molecular Pathology Laboratory (AMPL), Singhealth for human CD3 (Agilent Technologies), and B7-H3 (Cell Signaling Technology) staining and grading by a qualified pathologist (Ei Ei Thit).

### Cell Lines

NALM-6, DLD-1, HT29, SW-480, NCI-H1299, NCI-H596, NCI-H23, NCI-N87, MDA-MB-231, and MDA-MB-468 cells were purchased from ATCC. MKN-45 cells were purchased from DSMZ-German Collection of Microorganisms and Cell Cultures GmbH. RD114 packaging cell line was purchased from BioVec Pharma. Phoenix-ECO packaging cell line was purchased from ATCC. The stable RD114 retrovirus packaging cell line that produces GFP-Firefly Luciferase (GFP-FFluc) virus particles was a kind gift from Dr Masataka Suzuki (Baylor Center for Gene Therapy, Baylor College of Medicine).

Cell lines were frozen down as soon as possible after initiation of culture from ATCC stock. Cell lines were not authenticated but were used in early passages in all experiments (within 6–12 passages after initiation of culture from ATCC stock). All cell lines were tested with MycoAlert Mycoplasma Detection Kit (Lonza) biweekly or one day prior to freezing or experimentation. Cells found to be contaminated with *Mycoplasma* were treated with Plasmocure ™ (InvivoGen) as per manufacturer's instructions and retested for *Mycoplasma* 1 week after completion of treatment.

All cells were grown in complete media as recommended by cell supplier and maintained in a humidified atmosphere containing 5% CO_2_ at 37°C.

### Library Generation, Screening, and Identification of B7-H3–specific VHHs

To construct the VHH library, a naïve male llama was immunized with recombinant human B7-H3 (Sino Biological) at 100 µg per injection for a total of 6 injections. Total RNA was then isolated from peripheral blood lymphocytes and cloned into phagemid pMECs. The recombinant plasmids were then transformed into electrocompetent TG1 *Escherichia coli* cells to generate a VHH library of 10^9^ transformants that was further characterized by PCR and sequencing analyses. TG1 transformants were subsequently infected with the M13K07 helper phage to amplify and repackage the library for biopanning.

At each round of biopanning, the phage library was first incubated with unloaded streptavidin beads to remove nonspecific binders and the subtracted phage library was then incubated with streptavidin beads precoated with recombinant B7-H3 protein, before washing with PBST (PBS + 1% Triton X-100). Bound phages were then eluted with 0.1 mol/L triethylamine, amplified by infecting TG1 cells and used for next round of biopanning. Sequential rounds of biopanning were carried out with increasing stringency by reducing the concentration of biotinylated recombinant B7-H3 protein coated on streptavidin beads and increasing the frequency of washes.

Screening for B7-H3–specific VHHs was done by binding ELISA. First, individual phage-infected TG1 colonies were picked before induction for VHH expression using 1 mmol/L IPTG. Cell pellets of TG1 cultures were then incubated with TES (Tris/EDTA/sucrose) buffer. Cell suspension was then spun down and the supernatant containing the periplasmic extract was used for the binding ELISA. ELISA plates (Corning) were coated with 5 µg/mL neutravidin to capture biotinylated B7-H3 and bound VHHs were detected using an anti-HA antibody. Phagemids were then isolated from positive binders for sequencing.

### Expression and Characterization of Recombinant VHH

For large-scale expression of VHH, the genes were cloned into the pHEN6 expression vector and subsequently transformed into WK6 *E. coli* cells. Periplasmic extract was then prepared as described previously and VHH was further purified by affinity chromatography using nickel beads. To generate the VHH-Fc construct, VHH sequences were cloned into the pTT5 expression vector upstream of an IgG1-CH2CH3. Subsequently, HEK2936E cells were transiently transfected and recombinant VHH-Fc was purified from the culture supernatant using Protein G beads. Pooled fractions were then concentrated, and buffer exchanged into PBS pH 7.4 or MES pH 6.0.

Binding kinetics to recombinant B7-H3 was assessed by surface plasmon resonance (SPR) using Biacore T200 (Cytiva). Briefly, rabbit anti-VHH antibodies (Genscript) were immobilized on CM5 chips and purified VHHs were then captured by the anti-VHH antibodies. For initial screening, 100 nmol/L of recombinant B7-H3 was used to assess binding off-rates. For multi-cycle kinetic analysis, recombinant B7-H3 was injected at concentrations between 1 and 1,000 nmol/L in a, 2- or 3-fold dilution series. A reference channel with no VHH captured was used to correct for bulk effect and nonspecific binding while a blank run (no antigen flowed) was used to correct for surface stability. The double referenced sensorgrams were fitted with the Langmuir (1:1) binding model to obtain the association ka, dissociation kd, and equilibrium constant K_D_, and the closeness of fit was evaluated with the *χ*^2^ value.

Binding to cell-expressed B7-H3 was done with various cell lines. Murine CT26 and B7-H3 knockout human MKN7 cells were transfected to express murine B7H3-GFPSpark or human (4-Ig) B7H3-GFPSpark fusion proteins with their respective pCMV3 vectors (Sino Biological), using FuGene 6 transfection reagent (Promega). Transfection was then assessed by flow cytometry and fluorescence microscopy.

### Plasmid Constructs and Retrovirus Vectors Production

The B7-H3 human CAR construct consisted of the B7-H3–targeting VHH (P2A5 clone) inserted into a pSFG retroviral vector upstream of a 4-1BB–derived spacer, followed by human CD28 transmembrane domain, a human CD28 and CD3ζ signaling domains. A truncated form of the B7H3.CAR consisted only of the extracellular and transmembrane domain. In the B7-H3 murine CAR (B7H3 mCAR), the human CD28 transmembrane and signaling domains, and CD3ζ domain were replaced with their murine homologs, while keeping the antigen recognition anti-B7-H3 VHH domain and the 4-1BB spacer. On the basis of published work (22) indicating superior fitness in murine T cell–expressing CARs with reduced number of Immunoreceptor Tyrosine-based Activation Motif (ITAM) domains, we also inactivated the second and third ITAMs of the murine CD3ζ molecule by site-directed mutagenesis (Takara Bio).

Retrovirus vectors carrying the B7-H3–targeting human and murine CARs were produced in RD114 (BioVec Pharma) or Phoenix Eco (ATCC) packaging cell lines respectively, by transient transfection using PEIpro transfection reagent (Polyplus). Media containing retroviral vectors were harvested 48 and 72 hours posttransfection and concentrated using RetroX Concentrator (Takara Bio) before snap frozen and stored at −80°C.

### Generation of B7-H3–targeting Human and Murine CAR T Cells

Human peripheral blood mononuclear cells (PBMC) collected from consented healthy donors were purchased from HemaCare. Human B7H3.CAR T cells and B7H3.CAR EBVSTs, were generated on the basis of established protocols (23).

For murine CAR T cells, splenic T cells were isolated from congenic CD45.1 mice using the EasySep Mouse T Cell Isolation Kit (Stemcell Technologies) and activated with the Dynabeads Mouse T-Activator CD3/CD28 Kit (Thermo Fisher Scientific) for one day. Activated mouse T cells were transduced with retrovirus vectors encoding the B7H3 murine CAR transgene on days 1 and 2 after cell activation or left untransduced (UT). Cells were maintained in mouse T-cell medium containing 100 IU/mL human IL2 (R&D Systems) from activation till 2 days posttransduction and subsequently, 10 ng/mL human IL7 plus IL15 (R&D Systems) till harvest on day 3 posttransduction.

All human and murine T cells were cultured in Grex culture vessels (Wilson-Wolf) with frequent media and cytokines change in 5% CO_2_ at 37°C. Cell transduction efficiency and phenotyping of T cells were assessed by flow cytometry on day 2 or 3 posttransduction and at harvest. In all experiments, human and murine T cells were thawed and rested overnight in their respective complete medium containing 10 ng/mL human IL7 plus IL15. After recovery, viability and counts was assessed using Trypan Blue (Sigma-Aldrich) and hemocytometer counting.

### EBV Specificity of EBVSTs

The EBV specificity of EBVSTs in the product may be assessed by stimulating cells with pooled EBV peptides (JPT Peptide Technologies) in the presence of CD28 and CD49d antibodies (BD Biosciences). Medium alone (no peptide) and cells treated with human immunodeficiency virus (HIV) peptides (JPT Peptide Technologies) serve as negative controls. After overnight incubation in the presence of monensin and brefeldin A (BD Biosciences), T cells are stained with live-dead stain (Thermo Fisher Scientific), CD3, CD4, CD8 antibodies, fixed and permeabilized using BD Cytofix/Cytoperm, and stained with Allophycocyanin (APC)- or Phycoerythrin (PE)-conjugated IFNγ and TNFα antibodies (all reagents from BD Biosciences) followed by flow cytometry analysis.

### Gene Knockout of B7-H3 in Cell Lines

Single-guide RNAs targeting sequences 5′-CTGGTGCACAGCTTTGCTGA-3′, 5′GTGCCCACCAGTGCCACCAC-3′, and 5′-TGCCCACCAGTGCCACCACT-3′ (Integrated DNA Technologies, Inc and Synthego) were incubated with Cas9 (Integrated DNA Technologies, Inc) for 10 minutes to form RNP complex. Cell lines were washed with PBS and briefly incubated with RNP complex before electroporation using the 4D-Nucleofector (Lonza). Knockout efficiencies were assessed using flow cytometry and B7-H3–negative cells were selected by FACS using the BD Influx Cell Sorter (BD Biosciences).

### Real-Time Cytotoxicity and Serial Killing Potency Assays

All cytotoxicity and serial killing assays were carried out using the xCELLigence Real-Time Cell Analysis System (Agilent Technologies) following manufacturer's manual (Agilent Technologies).

### B7H3 CAR Activation and Competition Assay with Soluble B7-H3

UT and B7H3.CAR EBVSTs were stimulated with either the 4-Ig or 2-Ig form of recombinant soluble or plate-coated B7-H3 (Acro Biosystems). Cells were then assayed for intracellular IFNγ and TNFα and cell surface staining of CD25. For CD25 staining, cells were harvested 2 days poststimulation for flow cytometry analysis. For intracellular staining, GolgiSTOP and GolgiPlug were added to cells 1 hour poststimulation, and after an overnight incubation, cells were harvested for intracellular cytokine staining and flow cytometry analysis.

For competition assay, cytotoxicity of B7H3.CAR EBVSTs against NCI-N87 or NCI-H1299 was measured using the xCELLigence Real-Time Cell Analysis System (Agilent Technologies) in the presence of varying concentrations of soluble B7-H3 over a period of 48 hours.

### Mice

Breeder pairs of NSG-(K^b^ D^b^)^null^ (IA^null^) mice (NSG-MHC I/II DKO; RRID 025216) were purchased from Jackson Laboratory and bred at InVivos (Singapore) through contract breeding. Wildtype (WT) C57BL/6J (CD45.2; RRID 000664), congenic C57BL/6 (CD45.1; RRID 002014), and NOD-scid IL2Rgnull-3/GM/SF (NSG-SGM3; RRID 013062) were purchased from The Jackson Laboratory.

All mice were bred and kept under pathogen-free conditions in Biological Resource Centre, Agency for Science, Technology and Research, Singapore (A*STAR). All experiments and procedures were approved by the Institutional Animal Care and Use Committee of A*STAR, in accordance with the guidelines of the Agri-Food and Veterinary Authority and the National Advisory Committee for Laboratory Animal Research (NACLAR) of Singapore.

### Colorectal Cancer, Non–small Cell Lung Cancer, TNBC, and Gastric Cancer Cell Line–derived Xenograft Mouse Models

In the colorectal cancer model, 5 × 10^6^ of HT-29 or 2 × 10^6^ SW480 cells were subcutaneously injected into right flank of NSG-MHC I/II DKO mice. In the non–small cell lung cancer (NSCLC), TNBC, and gastric cancer models, 2 × 10^6^ NCI-H1299, 5 × 10^6^ MDA-MB-468, or 2 × 10^6^ of NCI-N87 cells were subcutaneously injected into right flank of NSG-MHC I/II DKO mice, respectively. Tumor sizes were measured using callipers and tumor volume was calculated by the formula: Volume = (width)^2^ × length/2. When tumors become palpable (100–200 mm^3^) between days 7 and 16, mice were randomized into treatment groups, stratified by tumor volume. 5 × 10^6^ of UT or B7H3.CAR EBVSTs were injected into mice intravenously while untreated mice served as controls. Physical examination, tumor, and body weight measurements were carried out twice a week until endpoint when tumors in control mice reach 1,000 mm^3^ in size. When mice were sacrificed, blood, spleen, liver, lungs, and tumor were collected for endpoint flow cytometric analysis.

With the exception of tumors and lungs which were dissociated into single-cell suspensions using Human Tumor and Mouse Lungs Dissociation Kits (Miltenyi Biotec), all other organs were processed using mechanical disruption.

All single-cell suspensions were stained with viability dye and antibodies to camelid VHH, anti-mouse CD45, anti-human CD45, CD3, CD4, CD8, PD-1, TIM-3, LAG-3, and B7-H3 for flow cytometry with addition of Countbright Absolute counting beads (Thermo Fisher Scientific) to enumerate viable cell populations.

### Breast Cancer and NSCLC Patient-derived Xenograft Mouse Model

Breast cancer BC370.1 and NSCLC L19130721 patient-derived xenograft (PDX) were subcutaneously injected into the right flank of NOD-*scid* IL2Rgamma^null^ (NSG) mice. When tumors became palpable between days 32 and 42, mice were randomized into treatment groups, stratified by tumor volumes. A total of 5 × 10^6^ of UT or B7H3.CAR EBVSTs were injected into mice intravenously. Untreated mice served as controls. Physical examination, tumor, and body weight measurements were carried out twice a week until study endpoint. Four mice were sacrificed at day 17 for IHC analysis and were excluded from tumor and body weight measurements and survival analysis.

### *In Vivo* Murine Safety Model in Immunocompetent Mice

B16F10 wild-type (B16F10-WT) cells were retrovirally transduced with human B7-H3 construct to create the B16F10-hB7H3 murine tumor cell line that expresses human B7-H3. A total of 5 × 10^5^ B16F10-hB7H3 cells were subcutaneously injected into the right flank of WT C57BL6/J mice. When tumors become palpable, mice were irradiated at 5 Gy. Three days after irradiation, mice were randomized into treatment groups and intravenously injected with 10 × 10^6^ UT or B7H3.mCAR T cells generated from congenic CD45.1 splenocytes. Untreated mice served as controls. Physical examination, tumor, and body weight measurements were carried out twice a week until endpoint when tumors in control mice reach 1,000 mm^3^ in size. When mice were sacrificed, blood, spleen, liver, lungs, tumor, brain, and bone marrow were harvested for endpoint flow cytometric and pathology evaluation. Cells were stained with viability dye and antibodies to camelid VHH, mouse CD45.1, CD45.2, CD11b, CD11c, Gr1, CD19, NK1.1, CD3, CD4, CD8, and B7-H3, with addition of Countbright Absolute counting beads (Thermo Fisher Scientific) for flow cytometry analysis.

### Hematoxylin and Eosin Staining and Pathology Evaluation

Harvested mouse organs and tumors were immediately fixed in 10% neutral buffered formalin solution (Sigma-Aldrich) for 24–48 hours before paraffin embedding. Samples were sent to AMPL, A*STAR for hematoxylin and eosin (H&E) staining. Briefly, the tissues were dehydrated in an ascending series of ethanol, cleared with xylene, and then embedded in paraffin wax. A total of 5-µm-thick sections were cut and placed onto glass slides. The slides were dewaxed in xylene and hydrated with descending series of ethanol before being stained with H&E stain. Pathology evaluation was performed by a qualified pathologist (R. Rajarethinam).

### *In Vitro* Safety Assays with PBMCs

PBMC were stimulated with GMCSF (Miltenyi Biotec), IFNγ (Miltenyi Biotec), TNFα (Miltenyi Biotec), lipopolysaccharide (Sigma-Aldrich), or media alone at the indicated concentrations before staining for B7-H3 expression.

In coculture experiments of PBMCs and effector T cells, PBMC were stimulated with above cytokines or media alone for one day before cytokines were washed off. PBMCs were further cultured for 2 days with CellTrace Violet-labelled UT or B7H3.CAR EBVST before analysis by flow cytometry. Similarly, monocytes purified from PBMCs using CD14 microbead (Miltenyi Biotec) were incubated with CellTrace Violet-labeled allogeneic UT EBVST or B7H3.CAR EBVST for 2 days before analysis by flow cytometry.

### *In Vitro* Safety Assays with Hematopoietic Stem and Progenitor Cells

CD34^+^ hematopoietic stem and progenitor cells (HSPC) were stimulated with 10 ng/mL each of Flt3-ligand (FLT3L), stem cell factor (SCF), and thrombopoietin (TPO; all from Miltenyi Biotec) for the specified durations before evaluation for B7-H3 expression.

To investigate possible cytotoxicity of B7H3.CAR T cells against HSPCs, stimulated and unstimulated CD34^+^ HSPCs were cocultured with UT or B7H3.CAR EBVSTs at an effector:target (E:T) ratio of 1:1 for 24 hours. HSPC subsets were stained with viability dye and antibodies to CD34, CD133, CD45RA, CD38a, and CD10 before flow cytometric analysis.

Erythroid and myeloid developmental potential were assessed using the StemMACS HSC-CFU Assay kit (Miltenyi Biotec) and colony types were identified as per the manufacturer's protocol.

### Evaluation of B7-H3 Expression on Reactivated Memory T Cells

PBMCs were activated with plate-coated anti-CD3/CD28 antibodies for 7 days to generate activated T cells (ATC), before pulsing with HIV or EBV pepmixes. Pulsed ATCs were then irradiated and cocultured with CellTrace Violet-labeled UT EBVST. GolgiSTOP and GolgiPlug (BD Biosciences) were added to cells 1 hour poststimulation. After an overnight incubation, cells were stained with viability dyes and antibodies for surface antigens before staining for intracellular IFNγ and TNFα. For analyses of cell proliferation and B7-H3 expression, cells were collected on day 2 and day 5 of culture for flow cytometry analysis.

### Generation MDSCs and *In Vitro* Assays

To generate MDSCs, CD14^+^ monocytes were cultured in human IL6 and GMCSF for 7 days (24). MDSCs were harvested on day 7 and stained with viability dye and antibodies against CD14, CD11b, CD33, CD15, CD66b, HLA-DR, and B7-H3 before staining for intracellular IL10, TGFβ1, and iNOS.

For MDSC targeting experiments, UT and B7H3.CAR EBVSTs were first labeled with CellTrace Violet (Thermo Fisher Scientific) before overnight incubation with allogeneic MDSCs in the stated E:T ratios. For assessment of suppressor function, CellTrace Violet-labeled UT and B7H3.CAR EBVSTs were cocultured with allogeneic MDSCs on anti-CD3/CD28-coated plate for 6 days before flow cytometry analysis. Proliferation was assessed on the basis of percentage of cells with CellTrace Violet dilution. Proliferation index was calculated by normalizing proliferated cell percentages against proliferated cell percentages in no MDSC control condition.

### Assessment of CRS Model Using Humanized NSG-SGM3 Mice

To generate humanized mice, 1 × 10^5^ CD34^+^ cord blood cells were intravenously injected into sublethally irradiated female NSG-SGM3 mice. Four weeks later, humanization of mice was verified by staining for human CD45 on peripheral blood cells. A total of 5 × 10^6^ luciferase-expressing HT-29 cells or 2 × 10^6^ luciferase-expressing NALM-6 cells were intravenously injected into humanized mice. Tumor burden in both groups was tracked by tracking bioluminescence on the IVIS Lumina S5 Imaging System (PerkinElmer). Eighteen days after tumor cells injection, mice xenografted with HT-29 were randomized to receive 5 × 10^6^ UT EBVSTs, B7H3.CAR EBVSTs or left untreated. Mice xenografted with NALM-6, were randomized to receive 5 × 10^6^ CD19.CAR T or left untreated. Physical examination, tumor, and body weight measurement were performed twice a week on all mice until endpoint at 7 days posttreatment. Blood serum was collected from mice on day 3 posttreatment for quantification of serum cytokines using LEGENDplex multiplex bead-based immunoassay (Human Inflammation Panel 1, BioLegend). Blood, spleen, liver, and bone marrow were harvested for endpoint flow cytometric analysis. Cells were stained with viability dye and antibodies to camelid VHH, mouse CD45, human CD45, CD3, CD14, CD19, CD11b, CD66b, CD11c, CD56, CD4, CD8, HLA-A3, B7-H3, with addition of Countbright Absolute counting beads (Thermo Fisher Scientific) for flow cytometry analysis.

### Flow Cytometry Analysis

All flow cytometry analyses were performed on the FACSymphony A3 cell analyzer (BD Biosciences) with FACSDiva software and data were analyzed in FlowJo v10.8.1 for Windows (Tree Star Inc).

### Statistical Analysis

Statistical analysis and visualization were performed using Prism 9 software for Windows (Graphpad Software Inc.). For comparisons between two groups, a two-tailed unpaired Student *t* test or was used. For comparisons in time courses or among three or more groups, one-way or two-way ANOVA with Tukey or Sidak posttest was applied where appropriate. For survival analysis, a Kaplan–Meier survival log-rank analysis was used.

### Data Availability Statement

The data generated in this study are available from the corresponding author upon request.

## Results

### B7-H3 Expression in Tumors is Higher Compared with Healthy Tissues

**FIGURE 1:**
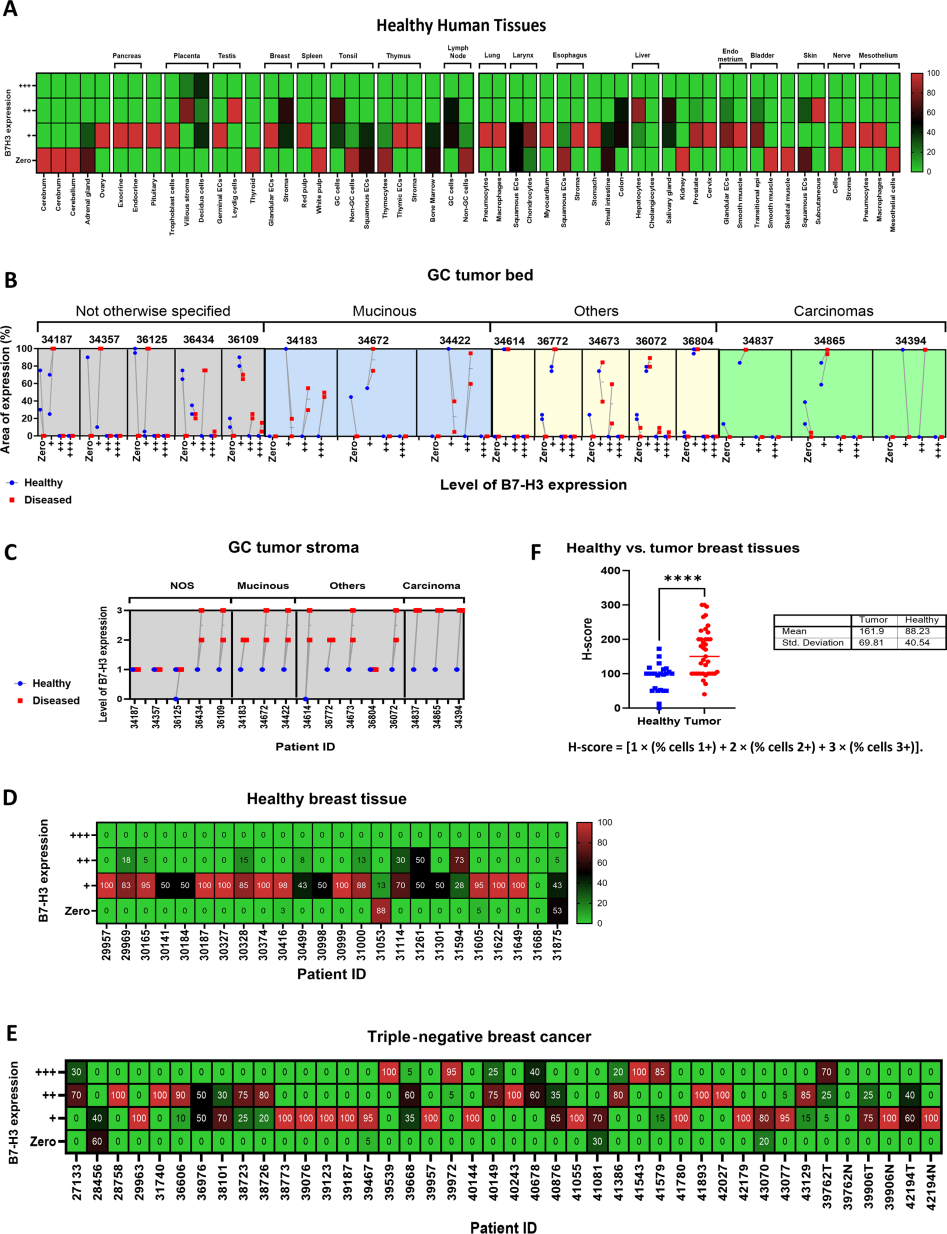
B7-H3 expression in tumors is higher compared with healthy tissues. **A,** Grading and pattern of B7-H3 expression in IHC staining of human normal tissues. **B,** Level and area of B7-H3 expression in gastric cancer tumor bed and adjacent healthy areas. **C,** Level of B7-H3 expression in gastric cancer tumor stromal. **D,** Level of B7-H3 expression in healthy breast tissues. **E,** Level of B7-H3 expression in TNBC. **F,** H-score for healthy versus tumor breast tissues. For F, groups were compared using Student unpaired *t* test. ****, *P* < 0.0001. Each ID or point on the graph represents a patient and error bars denote means ± SD where applicable.

We performed IHC staining using a commercially available anti-human B7-H3 antibody (Clone D9M2L) to examine B7-H3 expression on FFPE healthy human tissues microarray. Slides were subjected to a blinded analysis by an experienced pathologist (Ei Ei Thit). Analysis revealed B7-H3 cell surface expression to be absent on major organs such as brain, thyroid, kidney, bladder, and muscle, while other tissues including the adrenal gland, ovary pituitary gland, spleen, thymus, lungs, larynx, esophagus, stomach, pancreas, prostate, cervix, bone marrow, myocardium, nerve, and mesothelium exhibited only very weak or weak B7-H3 cytoplasmic with no surface staining. Moderate B7-H3 expression was observed in the testis, tonsils, colon, breast, lymph nodes, liver, endometrium, salivary gland, placenta, and skin (Fig. 1A; Supplementary Fig. 1A; Supplementary Table S1). Among these tissues, B7-H3 expression was largely confined to isolated cell types that included stroma and decidua cells in the placenta, stroma in the breast, germinal cells in the tonsils, germinal cells in the lymph nodes, hepatocytes in the liver, Leydig cells in the testis, and subcutaneous tissues in the skin (Supplementary Table S1). Of note, most healthy tissues with the exception of decidua cells in the placenta, Leydig cells in the testis, and skin subcutaneous tissues did not exceed H-scores of 200 (Supplementary Fig. S1B).

During IHC analysis of gastric tumor samples and corresponding healthy tissues to the tumors, we observed a significant upregulation of B7-H3 expression in the diseased areas compared with the adjacent healthy tissue (Supplementary Fig. S1C and S1D). These observations were consistent across different gastric cancer subtypes (Fig. 1B) and in the gastric cancer stromal compartment (Fig. 1C). Furthermore, B7-H3 was found to be strongly expressed in TNBCs while retaining a low expression in the contralateral or surrounding healthy breast tissues (Fig. 1D and E), further reflected in the significantly higher H-scores in TNBC samples compared with healthy breast tissues (Fig. 1F).

Consistent with data from other groups (3), our data indicate that B7-H3 expression is considerably higher in human solid tumors versus healthy tissues, providing a sound rationale for B7-H3 as a target for CAR T cells.

### Discovery and Identification of Lead B7-H3–targeting Nanobody

**FIGURE 2:**
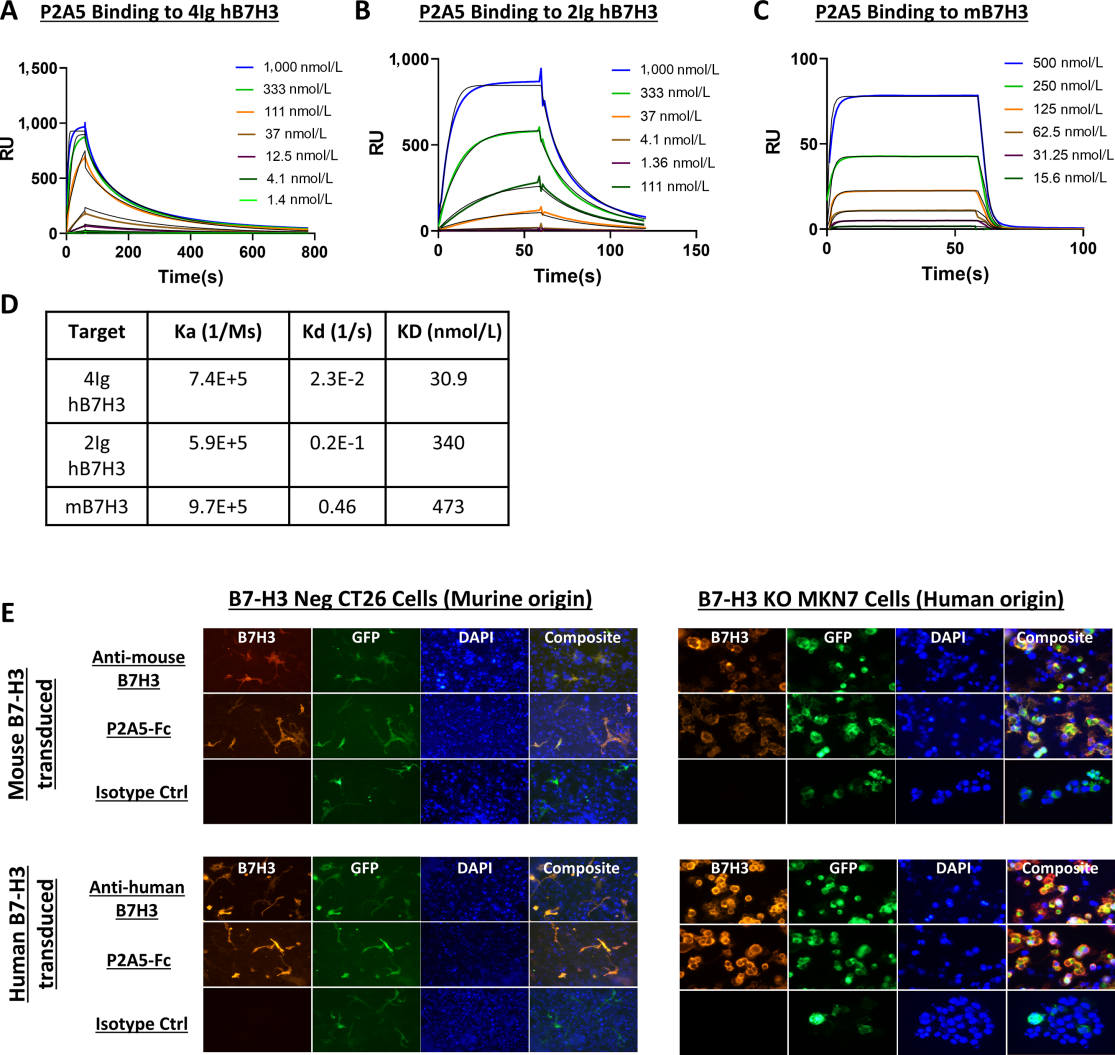
The P2A5 VHH binds to human and mouse B7-H3. SPR multi-cycle kinetic analysis of P2A5 binding to the 4-Ig (**A**) and 2-Ig (**B**) human B7-H3 isoforms, and to mouse B7-H3 (**C**). **D,** Binding kinetic parameters of P2A5 binding to the B7-H3 variants in A–C. **E,** Representative fluorescence microscopy images of P2A5 binding to human and mouse B7-H3. B7-H3–negative murine CT26 cells and human MKN7 cells knocked out for endogenous B7-H3 were transfected to express either murine B7-H3 or human 4-Ig B7-H3 and stained with positive control anti-mouse or anti-human B7-H3 antibodies, P2A5 as a VHH-Fc fusion molecule, or isotype control. GFP expression was used as an indicator of successful transfection.

Because the 4-Ig B7-H3 isoform is dominant in humans, VHHs specific for B7-H3 from an immunized llama library were isolated with repeated rounds of biopanning with recombinant 4-Ig B7-H3 proteins. Increasing stringency was applied by reducing the concentrations of target proteins from 100 nmol/L in the first round to 5 nmol/L in the third round. B7-H3 binders were screened by ELISA with phage eluted from rounds 2 and 3 (Supplementary Fig. S2A). Twenty unique leads were then expressed and further screened for binding off-rates to recombinant 4-Ig B7-H3 by SPR (Supplementary Fig. S2B) and for binding to endogenous B7-H3 expressed on the surface of HepG2 cells (Supplementary Fig. S2C). While most leads bound recombinant B7-H3 very strongly, only nine exhibited significant binding to B7-H3–expressing HepG2 cells. In a meta-analysis of 38 CAR T trials, CAR T cells utilizing antigen-binding domains with “moderate” affinity of K_D_ between 20 and 100 nmol/L produced the best clinical response (18%–75% complete and partial response; ref. 25). In addition, affinity is also an important factor that influences on-target, off-tumor toxicity. We thus focused on the clone with intermediate binding, P2A5, for further development. Using multi-cycle kinetic analysis by SPR, we measured the binding affinity of P2A5 to the 4-Ig and 2-Ig isoforms of human B7-H3 (Fig. 2A and B) and also to murine B7-H3 given its relatively high homology to the human 2-Ig isoform (Fig. 2C). We found that P2A5 binds 4-Ig B7-H3 with a dissociation constant K_D_ of around 30.9 nmol/L and to both the 2-Ig isoform and murine B7-H3 with the K_D_ of 340 nmol/L and 473 nmol/L, respectively (Fig. 2D).

B7-H3 glycosylation patterns have been described to be different between murine and human cells (26). To ascertain that the binding epitope of P2A5 on B7-H3 remains unaffected by the glycosylation in murine and human cells, we introduced either the human (4-Ig) or murine (2-Ig) B7-H3 molecules into a B7-H3–negative murine colon cancer cell line (CT26) and a B7-H3 knocked-out gastric adenocarcinoma cell line (MKN7). Successful transfection was determined by fluorescence microscopy detection of the GFP reporter gene. Transfected cells were then stained with either a commercial anti-mouse or anti-human B7-H3 antibody as positive control, P2A5 expressed in VHH-Fc format, or an isotype control, and visualized by fluorescence microscopy. We observed that P2A5 bound to murine and human B7-H3 expressed on both human MKN7 cells and murine CT26 cells (Fig. 2E). Hence P2A5 is cross-reactive to both murine and human B7-H3 even when expressed on cells from different species of origin.

### Generation, Characterization, and Validation of B7H3.CAR EBVSTs

**FIGURE 3:**
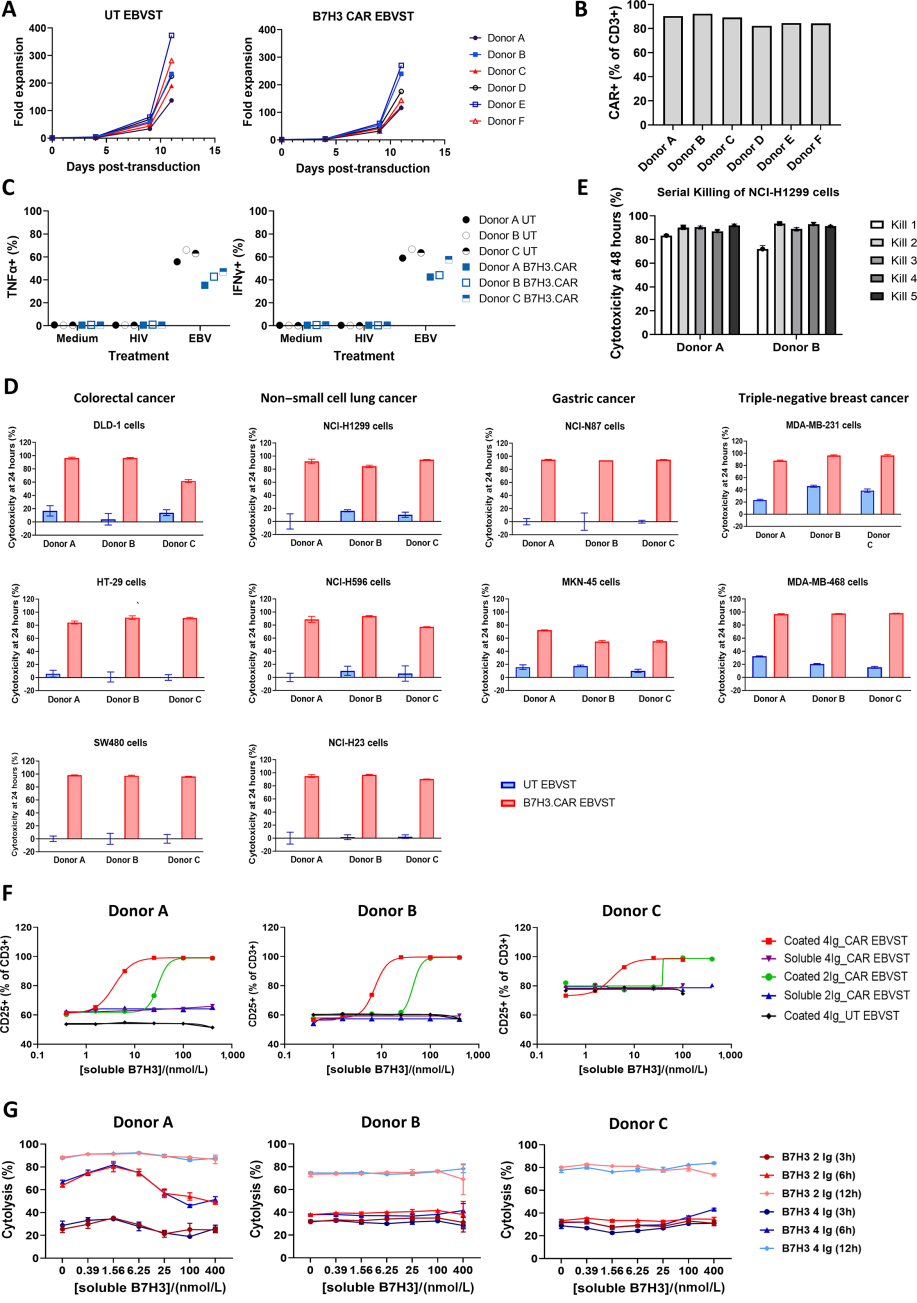
Generation and characterization of allogeneic B7H3.CAR EBVSTs. **A,** Fold expansion of EBVSTs following mock (UT) or B7H3.CAR transduction. **B,** CAR expression at final harvest. **C,** Percentages of EBVSTs expressing TNFα and IFNγ after overnight stimulation of cells with media, HIV or EBV pepmixes. **D,** UT and B7H3.CAR EBVST cytolysis of various B7-H3+ tumor cell lines after 24 hours of coculture at a 1:1 E:T ratio. **E,** Serial cytolysis of NCI-H1299 NSCLC cells by B7H3.CAR EBVSTs over five rounds of cocultures at a 1:2 E:T ratio. **F,** Cell surface staining of CD25 on EBVSTs after treatment with varying concentrations of plate-bound (coated) or soluble B7-H3 in 2-Ig or 4-Ig forms. **G,** Cytotoxicity of B7H3.CAR EBVST against NCI-N87 or NCI-H1299 cells at the indicated timepoints in the presence of varying concentrations of soluble 2-Ig or 4-Ig B7-H3. Data presented are from 6 (A and B), 3 (C, D, F, and G), or 2 (E) healthy donors and represent means ± SD where applicable.

We proceeded to clone the lead anti-B7-H3 VHH candidate P2A5 into a CAR, bearing a 4-1BB spacer domain, a CD28-derived transmembrane and intracellular cosignaling domain, and a CD3ζ activation domain (B7H3.CAR). A truncated CAR bearing only the spacer and transmembrane domains without the functional signaling domains (tB7H3.CAR) was used as a control (Supplementary Fig. S3A). To develop off-the-shelf allogeneic T cells for cancer treatment, we armored EBVSTs with the B7H3.CAR using an established protocol (ref. 23; Supplementary Fig. S3B). B7H3.CAR EBVSTs derived from 6 healthy donors expanded between 100- and 270-fold posttransduction (Fig. 3A), with B7H3.CAR expression consistently exceeding 80% in the final product (Fig. 3B). CD4^+^ cells with an effector memory (TEM) phenotype dominated the final B7H3.CAR EBVST product (Supplementary Fig. S3C and S3D).

To assess whether B7H3.CAR expression impacted endogenous TCR responses, we stimulated EBVSTs with pooled EBV pepmixes. B7H3.CAR EBVSTs production of intracellular IFNγ and TNFα in response to stimulation was robust, albeit at levels marginally lower than UT EBVSTs (Fig. 3C). Notably, nonspecific cytokine production in response to medium or irrelevant HIV peptides was low, indicating that the specificity and magnitude of responses to cognate antigens were maintained in the presence of the B7H3.CAR.

We next evaluated the specific cytotoxicity of B7H3.CAR EBVSTs against B7-H3+ cell lines and their B7-H3 knockout counterparts in real-time cytolysis assays. B7H3.CAR EBVSTs exhibited rapid and efficient killing of multiple colorectal cancer (DLD-1, HT-29, SW480), gastric cancer (NCI-N87, MKN-7, MKN-45), TNBC (MDA-MB-231, MDA-MB-468), and NSCLC (A549, NCI-H1299, NCI-H23, NCI-H596) cell lines, within 24 hours of coincubation (Fig. 3D). In contrast to WT NCI-N87 gastric cancer cell, knockout of B7-H3 in NCI-N87 tumor cells consistently abolished cytolysis and IFN production by B7H3.CAR EBVSTs (Supplementary Fig. S3E and S3F), showing that CAR-mediated killing and activation was dependent on B7-H3 expression. In addition, target killing of MKN-45 tumor cells was only seen with T cells bearing full-length B7H3.CAR with an intact intracellular signaling domain but not the truncated B7H3.CAR (Supplementary Fig. S3G). Together, these data show that tumor cell killing by B7H3.CAR EBVSTs is specific and contingent on B7-H3 recognition and full CAR activation. We further examined the ability of B7H3.CAR EBVSTs to perform serial killing of solid tumor cells in multiple rounds of coculture with NCI-H1299 NSCLC cells. B7H3.CAR EBVSTs from both donors consistently exhibited highly efficient cytotoxicity over five consecutive target encounters (Fig. 3E).

Given that elevated serum levels of soluble B7-H3 have been detected in patients with cancer (27), we next sought to determine whether B7H3.CAR EBVSTs could be activated by soluble B7-H3. Coincubation of B7H3.CAR EBVSTs with plate-bound or soluble B7-H3 revealed that B7H3.CAR EBVSTs upregulated CD25 (Fig. 3F) and secreted IFNγ/TNFα (Supplementary Fig. S3H) in response to stimulation by plate-bound B7-H3 but not soluble B7-H3. This suggests that B7H3.CAR EBVST activation requires CAR clustering, which can only be induced by a matrix of B7-H3 molecules but not with soluble monomers. To determine whether soluble B7-H3 monomers block B7H3.CAR EBVST targeting of B7-H3–positive tumor cells, we coincubated B7H3.CAR EBVSTs with tumor cells in the presence of soluble B7-H3 over 12 hours. Neither isoform of soluble B7-H3, even at high concentrations, affected cytolysis of NCI-N87 or NCI-H1299 tumor cells by B7H3.CAR EBVSTs (Fig. 3G).

### *In Vivo* Activity of B7H3.CAR EBVSTs Against B7-H3–positive Solid Tumors

**FIGURE 4:**
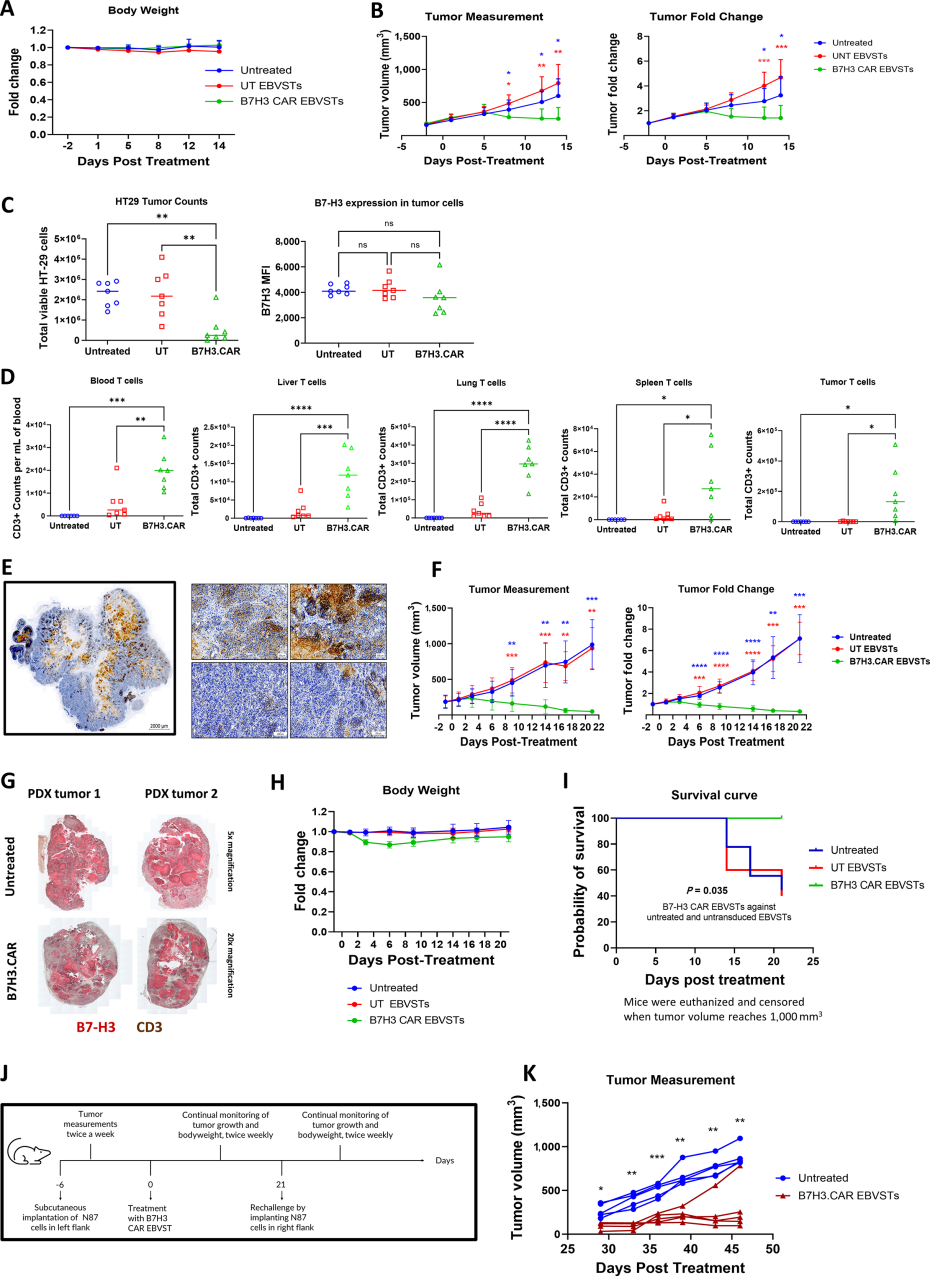
B7H3.CAR EBVSTs effectively target B7-H3–positive solid tumors *in vivo*. B7H3.CAR EBVST activity in a mouse model of HT-29 colorectal cancer (**A–D**). **A,** Body weight measurements posttreatment. **B,** Tumor volume (left) and fold change (right) after treatment. **C,** Endpoint absolute tumor cell counts and B7-H3 expression levels quantified by flow cytometry. **D,** Endpoint T-cell counts in blood, liver, lung, spleen, and tumor quantified by flow cytometry. B7H3.CAR EBVST activity was assessed in a breast cancer PDX model (**E–I**). **E,** IHC staining of B7-H3 on breast cancer PDX (left) with representative magnified images (right). Brown color represents positive B7-H3 staining. Blue color represents cell nucleus. **F,** Tumor volume (left) and fold change (right) after treatment. **G,** Representative IHC staining of B7-H3 and CD3 on tumors from 2 B7H3.CAR EBVST-treated mice and 2 untreated mice. Red color represents positive B7-H3 staining. Brown color represents positive CD3 staining. **H,** Body weight measurements posttreatment. **I,** Kaplan–Meier log-rank survival curve. Mice were euthanized and censored when tumor volume reaches 1,000 mm^3^. **J,** Experimental scheme to evaluate B7H3.CAR EBVST persistence by performing tumor rechallenge on contralateral flank of mice that had previously cleared NCI-N87 gastric tumors after B7H3.CAR EBVST treatment. **K,** Volume of NCI-N87 tumors on B7H3.CAR EBVST-treated mice after rechallenge and on control untreated mice. Each curve on the graph represents a mouse. Data comprise of 6–7 (A–D), 9–10 (E–I), and 5 (K) mice in each treatment arm. For A, B, F, H, and K, groups were compared using two-way ANOVA with Tukey test for multiple comparisons test. Comparison and *P* values between untreated versus UT EBVSTs, untreated versus B7H3.CAR EBVSTs, and UT versus B7H3.CAR EBVSTs groups is represented in black, blue and red, respectively. For C and D, groups were compared using one-way ANOVA with Tukey test for multiple comparisons test. *, *P* < 0.05; **, *P* < 0.01; ***, *P* < 0.001; ****, *P* < 0.0001. Error bars denote means ± SD where applicable.

To assess *in vivo* activity of B7H3.CAR EBVSTs against B7-H3–expressing colorectal cancer, we implanted immunodeficient NSG-MHC I/II DKO mice with HT-29 cells. Following tumor engraftment, mice were randomized to receive no treatment or treatment with UT or B7H3.CAR EBVSTs (Supplementary Fig. S4A). Body weights of mice were similar and stable across treatment groups posttreatment (Fig. 4A). While tumor growth continued unabated in mice that received no treatment or UT EBVSTs, treatment with B7H3.CAR EBVSTs induced significant tumor regression (Fig. 4B). Indeed, endpoint flow cytometry analysis corroborated these data with a significantly smaller population of viable HT-29 cells in tumors of mice treated with B7H3.CAR EBVSTs (Fig. 4C, left). Of note, the few HT-29 cells remaining in tumors of B7H3.CAR EBVST-treated mice retained high expression of B7-H3, indicating the absence of tumor antigen downregulation and escape (Fig. 4C, right). Compared with UT EBVSTs, significantly more B7H3.CAR EBVSTs were present in the blood, liver, lung, spleen, and tumors of mice (Fig. 4D). The significant presence of intratumoral B7H3.CAR EBVSTs, along with increased expression of activation-induced PD-1 with TIM-3 (Supplementary Fig. S4B), provides evidence that B7H3.CAR EBVSTs migrated efficiently into tumor sites where they exerted potent antitumor activity. Expression of LAG-3 on B7H3.CAR EBVSTs was universally low in all sites. Additional *in vivo* studies to assess activity of B7H3.CAR EBVSTs against B7-H3–expressing gastric cancer, TNBC, NSCLC and a second colorectal cancer xenografts demonstrated similarly promising efficacy and tolerability outcomes (Supplementary Fig. S4C). In the NCI-N87 gastric cancer model, we observed once more that intratumoral B7H3.CAR EBVSTs upregulated PD-1 and TIM-3 but not LAG-3 expression (Supplementary Fig. S4D), suggesting activation within tumor sites and robust antitumor activity.

To examine *in vivo* activity of B7H3.CAR EBVST in systems that more accurately represent biology of human cancers, we used a breast cancer PDX characterized by a heterogenous moderate to high B7-H3 expression pattern, with discrete regions displaying varying levels of B7-H3 protein (Fig. 4E). Upon establishment of the PDX, mice were randomized to receive no treatment or treatment with UT or B7H3.CAR EBVSTs. While tumor growth continued to increase in mice that received no treatment or UT EBVSTs, treatment with B7H3.CAR EBVSTs induced dramatic regression with barely detectable tumors by study endpoint (Fig. 4F). Consistent with these observations, endpoint flow cytometric analysis revealed significantly fewer viable tumor cells in mice treated with B7H3.CAR EBVSTs compared with mice in the other arms (Supplementary Fig. S4E). When we sacrificed 2 representative mice from the B7H3.CAR EBVST and untreated groups for IHC staining of PDX tumors, we observed a dramatic infiltration of human T cells in PDX tumors from B7H3.CAR EBVST-treated mice. The scant PDX tumor cells remaining in B7H3.CAR EBVST-treated mice retained high expression of B7-H3, supporting that there was no antigen escape arising from CAR T-cell therapy (Fig. 4G). Treatment with B7H3.CAR EBVST was well tolerated with only transient and mild body weight decreases observed in these mice (Fig. 4H). Notably, B7H3.CAR EBVST treatment significantly improved survival compared with untreated and UT EBVST treatment (Fig. 4I).

We extended our studies to a second NSCLC PDX that displayed relatively weak B7-H3 expression (Supplementary Fig. S4F). Despite lower B7-H3 positivity, the administration of B7H3.CAR EBVSTs effectively inhibited tumor growth in 4 of 8 mice, in contrast to the uncontrolled tumor growth seen in all untreated and UT EBVST-treated mice (Supplementary Fig. S4G). B7H3.CAR EBVST treatment significantly prolonged survival by a median of 47.5 days compared with 36.5 and 35.5 days for the untreated and UT EBVST conditions, respectively (Supplementary Fig. S4H). Unfortunately, mice responding to B7H3.CAR EBVST treatment developed xenogeneic GVHD, characterized by weight loss and lethargy, and the experiment had to be terminated at day 49.

Finally, as B7H3.CAR EBVSTs recovered from both HT-29 colorectal cancer and NCI-N87 gastric cancer cell line–derived xenografts displayed increased expression of PD-1 and/or TIM-3 (Supplementary Fig. S4B and S4D), which are also markers associated with T-cell exhaustion, we sought to evaluate long-term persistence and functionality of B7H3.CAR EBVSTs *in vivo*. To address this, we subjected mice that had successfully eradicated primary NCI-N87 gastric cancer tumors following B7H3.CAR EBVST treatment to a second tumor challenge on the contralateral flank (Fig. 4J). In contrast to control untreated mice which all experienced tumor engraftment, 4 of 5 mice that received earlier administration of B7H3.CAR EBVSTs rejected the second tumors (Fig. 4K). These findings demonstrate that B7H3.CAR EBVSTs can persist *in vivo* and have a robust capacity to eliminate tumors upon rechallenge.

### CAR T-cell Targeting of Murine B7-H3 in Immunocompetent Mice is Associated with Minimal On-Tumor Off-Target Toxicity

On the basis of the observation that P2A5 VHH is cross-reactive to mouse B7-H3, we established an immunocompetent murine model to evaluate potential toxicity of the B7H3.CAR. ATCs from congenic CD45.1 mouse splenocytes were retrovirally transduced with the B7H3 murine CAR (B7H3.mCAR), achieving a CAR expression of 70% at harvest (Supplementary Fig. S5A). We assessed the cytotoxicity of B7H3.mCAR T against the target cell lines B16F10-WT, B16F10-overexpressing human B7-H3 (B16F10-hB7H3), and B16F10 overexpressing murine B7-H3 (B16F10-mB7H3). A dose-dependent cytolysis of B16F10-hB7H3 and B16F10-mB7H3 cells but not B16F10-WT cells was observed (Supplementary Fig. S5B), indicating that B7H3.mCAR T displayed specific and potent cytotoxic activity against both human and mouse B7-H3–expressing tumor cells.

**FIGURE 5:**
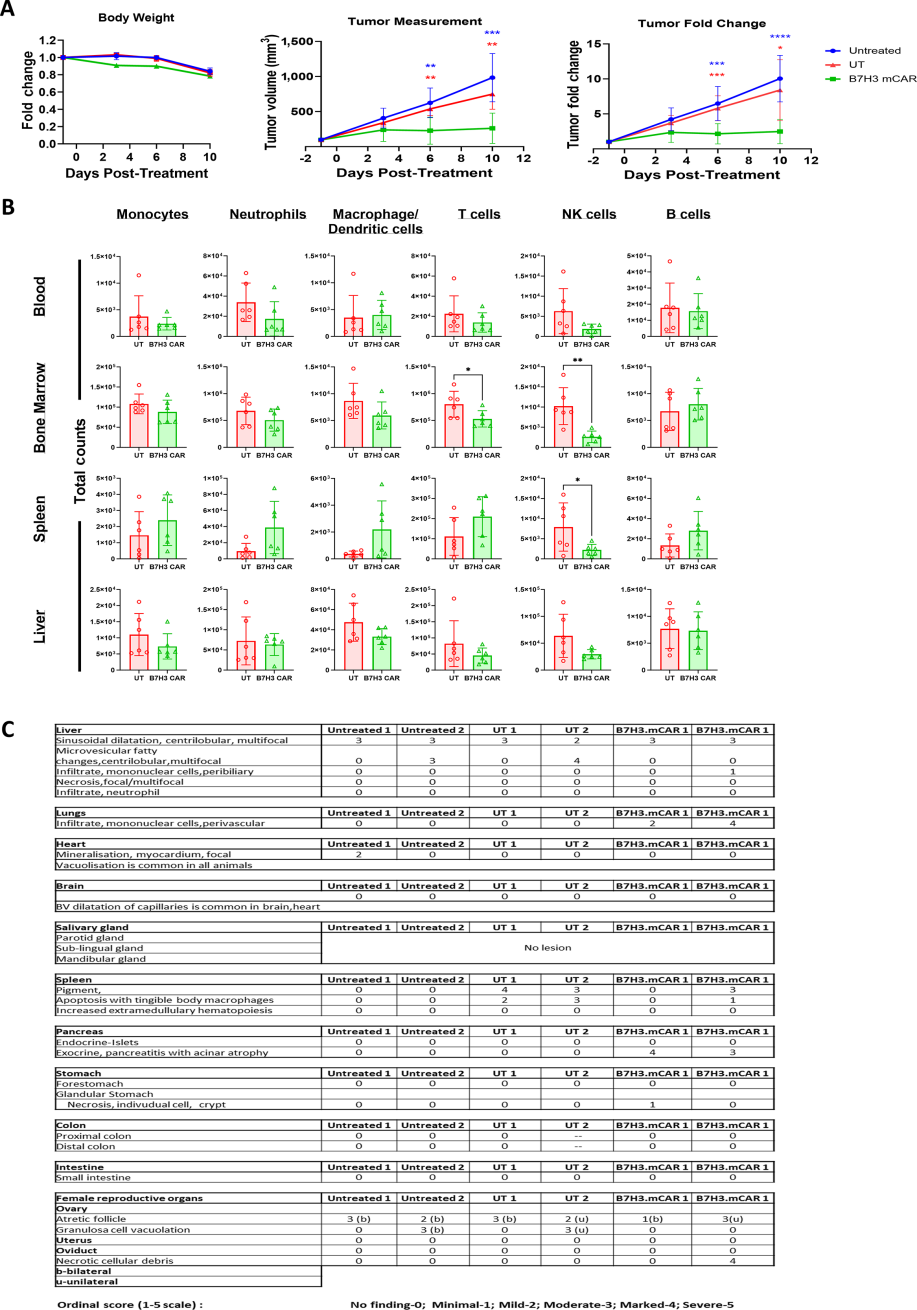
B7-H3.mCAR T cells exhibit little on-tumor off target toxicity in a syngeneic mouse model. **A,** Body weight (left), tumor volume measurements (middle), and fold change in tumor volumes (right) of B16F10-hB7H3 tumors after treatment. **B,** Endpoint murine immune cell counts in blood, bone marrow, spleen, and liver quantified by flow cytometry. Each point on the graph represents a mouse. **C,** Pathologic evaluation of organs from two B16F10WT-hB7H3 tumor-bearing C57BL/6J mice receiving UT or B7H3.mCAR T cells or left untreated. For A and B, data comprise of 6 mice in each treatment arm. For A, groups were compared using two-way ANOVA with Tukey test for multiple comparisons test. Comparison and *P* values between untreated versus UT T, untreated versus B7H3.CAR EBVSTs, and UT T versus B7H3.CAR EBVSTs groups is represented in black, blue, and red, respectively. For B, groups were compared using Student unpaired *t* test. *, *P* < 0.05; **, *P* < 0.01; ***, *P* < 0.001; ****, *P* < 0.0001. Error bars denote means ± SD where applicable.

When WT C57BL/6J mice were xenografted with B16F10-hB7H3 tumors (Supplementary Fig. S5C), tumors continued to grow in mice that received no treatment or UT T cells while B7H3.mCAR T-cell treatment significantly suppressed tumor growth (Fig. 5A, middle and right). Weight changes in mice treated with B7H3.mCAR T cells was similar to untreated and UT T cells (Fig. 5A, left), with none of the mice developing any signs of morbidity or weakness during daily follow-up. Endpoint analysis at day 10 revealed that mice receiving B7H3.mCAR T only experienced reduction of T cells in the bone marrow and natural killer (NK) cells in the bone marrow and spleen. No significant losses of other hematologic subsets were detected (Fig. 5B).

Two representative mice in each treatment arm further underwent a blinded histopathologic evaluation by an experienced pathologist (R. Rajarethinam). No remarkable histopathology differences were observed in the examined tissues between the three groups, with the exceptions of lungs and pancreas (Fig. 5C; Supplementary Fig. S5D). Grade 3–4 chronic pancreatitis with acinar atrophy was noted to be present in both animals which received B7H3.mCAR T cells, as were lymphocyte infiltration in perivascular areas of the lungs. The possible cause of pancreatitis in the current study is not known. Given the absence of any other observable lung pathology in the B7H3.mCAR T cell–treated mice and the known ability of B16 melanoma cells to produce lung metastases, we hypothesize that these lymphocytes represent B7H3.mCAR T cells migrating in response to micrometastases.

Taken together, we found no evidence of severe or widespread toxicities experienced by mice treated with B7H3.mCAR T cells.

### Targeting of Allogeneic Immune Cells by B7H3.CAR EBVSTs

To examine on-target off-tumor targeting of human immune cells by B7H3.CAR EBVST, we examined B7-H3 expression on PBMC subsets after stimulation with various inflammatory cytokines. B7-H3 expression on T, B, and NK cells remained low across various concentrations of GMCSF, IFNγ, TNFα (Supplementary Fig. S6A) and at IL7 and IL15 concentrations mimicking lymphodepletion conditions (Supplementary Fig. S6B). In contrast, GMCSF, which is frequently elevated in the serum of patients following CAR T-cell activation (28–30), induced up to 90% of monocytes to upregulate B7-H3 (Supplementary Fig. S6A).

**FIGURE 6:**
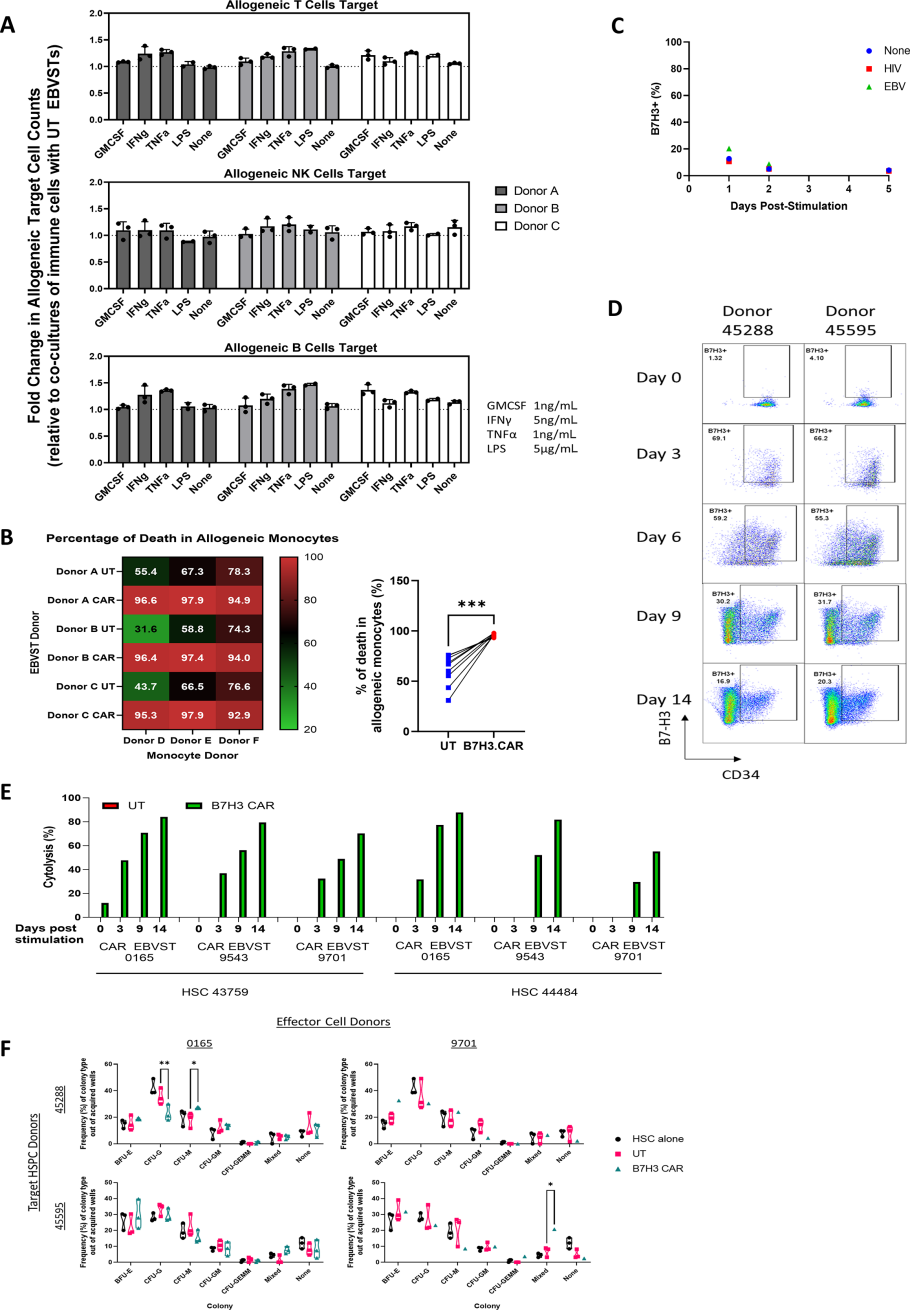
B7H3.CAR EBVSTs targets allogeneic monocytes and matured HSPCs but spares T, B, and NK cells as well as resting and early HSPCs. **A,** Cytolysis of allogenic T, NK, and B cells by UT and B7H3.CAR EBVSTs in the presence of various cytokines. Fold change in cell counts was calculated by normalizing allogeneic immune cell counts in presence of B7H3.CAR EBVSTs against that of UT EBVSTs. **B,** Percentage of death in allogeneic monocytes after 24 hours coculture at a 1:1 E:T ratio with UT or B7H3.CAR EBVSTs. **C,** B7-H3 expression on memory EBVSTs that have been reactivated with antigen-presenting cells pulsed with EBV, HIV, or no peptides. **D,** Flow plots representing B7-H3 expression in HSPC populations after the indicated days of stimulation with Flt3L, TPO, and SCF. **E,** Cytotoxicity of unstimulated and stimulated HSPCs after coculture with UT or B7H3.CAR EBVSTs. **F,** Erythroid and myeloid development potential of HSPCs alone, after coculture with UT or B7H3.CAR EBVSTs. Data in A and C are compiled from UT and B7H3.CAR EBVSTs generated from 3 donors cocultured with PBMCs or monocytes isolated from 3 other different donors. Data presented in C are representative of B7-H3 expression on reactivated EBVSTs from 2 donors. Data in D–F are compiled from UT and B7H3.CAR EBVSTs generated from 3 donors cocultured with HSPCs isolated from 2 other different donors. For B, groups were compared using Student unpaired *t* test. For F, groups were compared using one-way ANOVA with Tukey test for multiple comparisons test. *, *P* < 0.05; **, *P* < 0.01; ***, *P* < 0.001. Error bars denote means ± SD where applicable.

As B7H3.CAR EBVSTs were developed as an allogeneic cell therapy product, we next asked whether host immune cells might upregulate B7-H3 in the presence of allogeneic T cells, thereby becoming targets of B7H3.CAR EBVSTs. To address this, we cocultured PBMCs with allogeneic UT or B7H3.CAR EBVST in the presence of various cytokines (Supplementary Fig. S6C). Consistent with the minimal expression of B7-H3 detected in these cells (Supplementary Fig. S6D), no discernible loss in T, B, and NK cell populations was observed after coculture with B7H3.CAR EBVST (Fig. 6A). When we cocultured purified monocytes with allogeneic EBVSTs, we found that monocytes expressed exceptionally high levels of B7-H3 (Supplementary Fig. S6E). Coculturing of monocytes with allogeneic B7H3.CAR EBVST led to significantly higher death of target monocytes compared with UT EBVST cells (Fig. 6B).

As B7-H3 is upregulated on ATCs (28), we further examined whether memory T cells reactivated by their cognate antigens would upregulate B7-H3. To reactivate a memory T-cell population, we activated EBVSTs using a mixture of EBV peptides (Supplementary Fig. S6F), resulting in production of IFNγ and TNFα, and proliferation (Supplementary Fig. S6G). In the 5 days following stimulation, EBVSTs maintained low levels of B7-H3 (Fig. 6C), suggesting that B7H3.CAR EBVSTs would be unlikely to target memory T cells, even after antigen-specific activation of the latter.

To examine whether B7H3.CAR EBVSTs target allogeneic immune progenitor populations, we first assessed B7H3 expression on cord blood CD34^+^ cells from two donors after FLT3L, SCF, and TPO cytokine stimulation (29). In both donors, B7-H3 expression was undetectable on resting CD34^+^ HSPCs but progressively increased from day 3 to day 14 of stimulation, across all progenitor populations, albeit at B7-H3 levels considerably lower than NCI-N87 tumor cells (Fig. 6D; Supplementary Fig. S6H). Coculture of resting or stimulated CD34^+^ HSPCs with B7H3.CAR EBVSTs led to significant cytolysis of the more matured 9-day (mean ± SD of 55.88% ± 16.99%) and 14-day (mean ± SD of 76.43% ± 11.96%) stimulated HSPCs compared with 3-day (mean ± SD of 24.79% ± 20.03%) or unstimulated HSPCs (mean ± SD of 2.00% ± 4.89%; Fig. 6E). Further analysis of the HSPC populations revealed more pronounced reductions of the MPP and LMPP subsets (Supplementary Fig. S6I). To investigate whether B7H3.CAR EBVSTs can impact the development of early differentiated HSPCs, we performed colony-forming unit assays on HSPCs that had first been stimulated with cytokines for 3 days and subsequently cocultured with B7H3.CAR EBVSTs (Supplementary Fig. S6J). As coculture with B7H3.CAR EBVSTs resulted in significant cytolysis of 9 and 14 days stimulated HSPCs, we did not continue to examine whether any remaining HSPCs could develop into differentiated lineages. The erythroid and myeloid differentiation capacity of HSPCs remained unaffected by B7H3.CAR EBVSTs, evident by the lack of major reductions in mature progenitor subsets (Fig. 6F).

### B7H3.CAR EBVST Therapy is Associated with a Lower Risk of CRS as a Result of *In Vivo* Targeting of Allogeneic Myeloid Cells

We utilized an established humanized mouse model of CRS (30) to evaluate toxicities associated with B7H3.CAR EBVST treatment in solid tumors. As most of our knowledge on CRS associated with CAR T-cell therapy is drawn from treating acute leukemia with CD19 CAR T cells, we used this model as a benchmark for comparison.

Luciferase-expressing HT-29 or NALM-6 cells were injected intravenously into humanized mice to establish a metastatic colorectal cancer or acute leukemia model, respectively. When systemic tumor burden was sufficiently high (average radiance of more than 10^7^), mice xenografted with HT-29 cells were randomized to receive no treatment, UT EBVSTs or B7H3.CAR EBVSTs while mice xenografted with NALM-6 received no treatment or CD19.CAR T cells (Supplementary Fig. S7).

**FIGURE 7:**
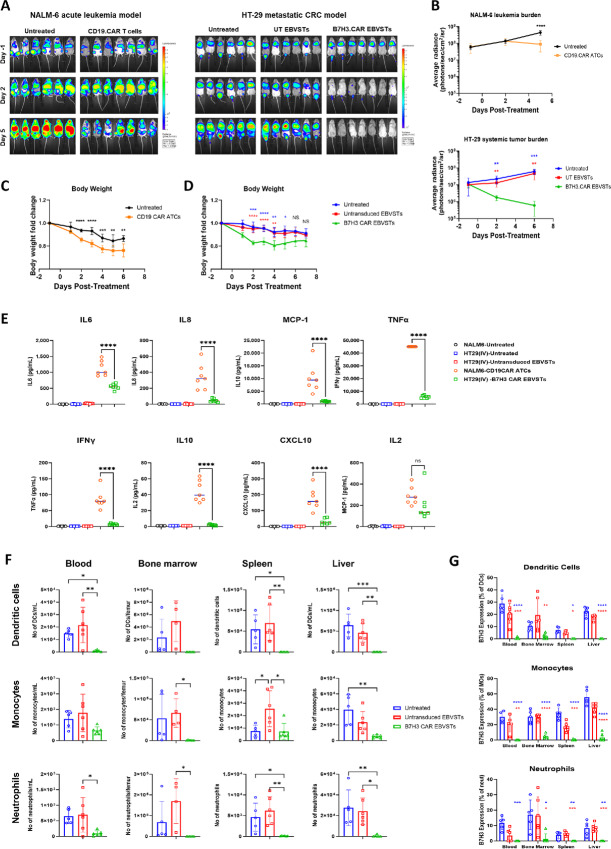
*In vivo* targeting of allogeneic myeloid cells by B7H3.CAR EBVSTs is linked to a lower risk of CRS. **A,** IVIS monitoring was performed to track tumor progression. Bioluminescence images of tumor burden in mice injected intravenously with NALM-6 (left) or HT-29 (right) at days −1, 2, and 5 posttreatment. **B,** Average tumor burden represented in mice of each treatment group injected with NALM-6 (top) or HT-29 (bottom). Body weight measurements of NALM-6 (**C**) or HT-29-injected (**D**) mice posttreatment. **E,** Serum levels of human cytokines and chemokines at 3 days posttreatment as quantified by multiplex bead immunoassay. **F,** Total counts of dendritic cells (top), monocytes (middle), and neutrophils (bottom) in blood, bone marrow, spleen, and liver of each treatment groups quantified by flow cytometry. **G,** B7-H3 expression on dendritic cells (top), monocytes (middle), and neutrophils (bottom) in blood, bone marrow, spleen, and liver after treatment, quantified by flow cytometry. Data comprise of up to 6–9 mice in each treatment arm. For B–D, groups were compared using two-way ANOVA with Tukey test for multiple comparisons test. Comparison and *P* values between untreated versus UT T, untreated versus B7H3.CAR EBVSTs and UT T versus B7H3.CAR EBVSTs groups is represented in black, blue, and red, respectively. For E–G, groups were compared using one-way ANOVA with Tukey test for multiple comparisons test. *, *P* < 0.05; **, *P* < 0.01; ***, *P* < 0.001; ****, *P* < 0.0001; ns, not significant. Each point on the graph represents a mouse and error bars denote means ± SD where applicable.

Both CD19.CAR ATCs and B7H3.CAR EBVSTs elicited good antitumor responses, evident from the lower tumor burden compared with the untreated or UT EBVST treatment groups (Fig. 7A and B). However, despite the disease control, CD19.CAR ATC-treated mice showed a rapid and unremitting weight loss of more than 20% by 4 days posttreatment (Fig. 7C). In contrast, B7H3.CAR EBVST-treated mice experienced a transient weight loss at 4 days posttreatment from which they quickly recovered (Fig. 7D). All CD19.CAR ATC-treated mice were euthanized at days 5–6 after treatment due to rapid health deterioration while B7H3.CAR EBVST-treated mice remained clinically well until study endpoint.

Serum levels of human cytokines including IL6, IL8, IL10, IL2, IFNγ, and TNFα in B7H3.CAR EBVST-treated mice were significantly lower than in CD19.CAR ATC-treated mice at 3 days posttreatment, suggesting that cytokine release following B7H3.CAR EBVST treatment was contained (Fig. 7E). Analysis of hematologic subsets in multiple organs of humanized mice at study endpoint revealed that myeloid cells such as neutrophils, monocytes, and dendritic cells were dramatically diminished in the B7H3.CAR EBVST-treated group (Fig. 7F), with reduction largely occurring in the B7-H3–expressing population (Fig. 7G). Altogether, this supports the notion that B7H3.CAR EBVSTs target allogeneic myeloid cells in a B7-H3–dependent manner and by diminishing these primary cellular mediators of CRS, attenuate the risk of CRS associated with CAR T-cell therapy.

### B7H3.CAR EBVSTs are Able to Target MDSCs

**FIGURE 8:**
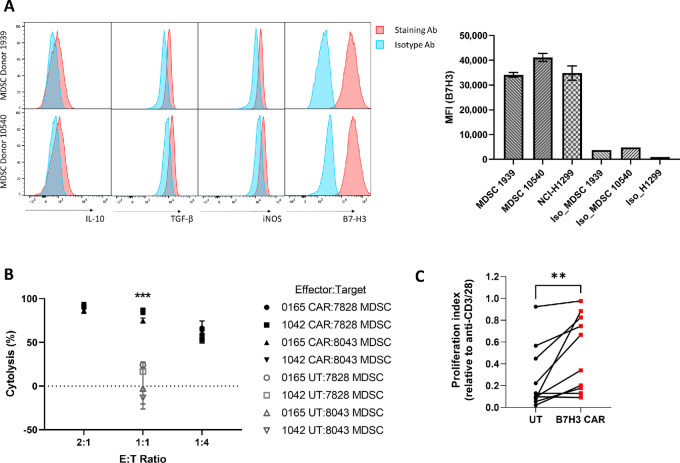
B7H3.CAR EBVSTs targets MDSCs. **A,** Flow cytometric histograms of IL10, TGFβ, iNOS, and B7-H3 in MDSCs generated from 2 healthy donors (left). Median fluorescence intensities (MFI) of B7-H3 expressed on MDSCs from 2 healthy donors and the NCI-H1299 cell line (right). **B,** Cytotoxicity of UT or B7H3.CAR EBVSTs against allogeneic MDSCs. Data compiled from coculture of effector EBVSTs generated from 2 donors and target MDSCs generated from 2 other donors. **C,** Proliferation of UT and B7H3.CAR EBVSTs after anti-CD3/CD28 stimulation in the presence of allogeneic MDSCs. Each datapoint represents proliferation from each E:T donor pair. Proliferation index was calculated by normalizing percentages of proliferated cells against that of the no MDSC control condition. For B and C, UT versus B7H3.CAR EBVSTs groups were compared using Student unpaired *t* test. *, *P* < 0.05; **, *P* < 0.01; ***, *P* < 0.001. Error bars denote means ± SD where applicable.

We noted that monocytes upregulate B7-H3 during inflammatory conditions (Supplementary Fig. S6A) and were targets for B7H3.CAR EBVSTs (Fig. 6B). To determine whether MDSCs, exhibiting characteristics akin to inflammatory monocytes, express B7-H3 and would thus be susceptible to targeting by B7H3.CAR EBVST, we first differentiated MDSCs from purified monocytes with GMCSF and IL6 for 7 days and stained for surface expression of B7-H3. MDSCs were found to express high levels of B7-H3, along with inhibitory molecules IL10, TGFβ, and iNOS (Fig. 8A). Notably, MDSCs expressed B7-H3 at levels comparable to the lung cancer cell line, NCI-H1299 (Fig. 8A). MDSCs were verified as immunosuppressive based on their dose-dependent inhibition of anti-CD3/28-induced T-cell proliferation (Supplementary Fig. S8). In contrast to the low levels of cytolysis seen in MDSCs cocultured with UT EBVSTs, B7H3.CAR EBVSTs killed MDSCs across varying cell ratios (Fig. 8B). To further investigate the effect of MDSCs elimination on T-cell function, we stimulated UT or B7H3.CAR EBVSTs with anti-CD3/CD28 in the presence of MDSCs. Although MDSCs hindered the proliferation of UT EBVSTs, their inhibitory effect on B7H3.CAR EBVSTs was significantly less pronounced, as indicated by the higher T-cell proliferation index (Fig. 8C). This indicated that targeting of B7-H3–expressing MDSCs by B7H3.CAR EBVSTs alleviated MDSC-induced inhibition of T-cell proliferation.

## Discussion

To overcome the bottlenecks that have hampered autologous CAR T-cell therapy, we utilized EBVSTs as our universal T-cell platform to develop off-the-shelf CAR T-cell therapy against solid tumors. Here, we demonstrate that our nanobody-based CAR engineered EBVSTs provide a tumor-agnostic strategy to target diverse types of B7-H3–positive solid tumors while overcoming some of the hurdles that have impeded clinical advancement of CAR T cells against such tumors.

Employing EBVSTs as CAR T-cell hosts offers several advantages. First, EBV infects about 90% of adults worldwide (31) with a notable enrichment in circulating EBV-specific effector CD4^+^ and CD8^+^ T cells that persists throughout life (32, 33). This suggests that EBVSTs can be readily extracted from the blood of EBV-exposed but otherwise healthy donors for scalable production. Second, the TCR repertoire of EBVSTs is greatly EBV focused and reduced in diversity compared with CD3-ATCs, thereby lowering the potential for alloreactivity (23, 34). This notion is reinforced by a lack of clinically worrying GVHD reported in multiple clinical trials using allogeneic EBVSTs (>300 recipients) and underscores the safe application of third party EBVSTs as an off-the-shelf cellular therapy (35–37), without the use of genetic editing. Third, different groups have detected significant populations of virus-specific T cells (VST), including EBVSTs, in human tumors across diverse cancer types (38–40), suggesting that VSTs are endowed with unique but yet-to-be-understood abilities to migrate into solid tumors. It would be interesting to explore whether the VST origin of B7H3.CAR EBVSTs would enhance their ability to traffic to and penetrate solid tumors compared with their CAR T-cell counterparts. Finally, EBV vaccination to stimulate endogenous TCRs has been advocated as a means to boost CAR armored EBVST expansion and function in patients (41, 42). This approach has been bolstered by recent strides in EBV vaccine delivery that have enabled effective and durable induction of EBV-specific T-cell responses (43).

Compared with hematologic malignancies, targeting solid tumors with CAR T-cell therapy presents a herculean set of challenges, including heterogeneous expression of cancer antigens within tumors, the paucity of tumor-restricted antigens which can increase the risk of on-target off-tumor toxicities, compromised T-cell survival in a hostile and immunosuppressive TME, and unknown ability of CAR T cells to infiltrate solid tumors (2, 44, 45). Our studies offer encouraging evidence that EBVSTs armored with B7-H3–targeting CARs are not only safe and efficacious against B7-H3–positive solid tumors but also address the longstanding challenges that have hampered the clinical development of CAR T cells in such indications. Using PDX models which recapitulate the complex biology and heterogeneity inherent to patient tumors, we demonstrated that our B7H3.CAR EBVSTs displayed good antitumor activity and significantly improved survival not only in a highly B7-H3–positive breast cancer PDX model, but also in a NSCLC PDX where B7-H3 expression was considerably weaker. It is noteworthy that there was no evidence of B7-H3 antigen escape occurring in tumors from B7H3.CAR EBVST-treated mice in all our CDX and PDX studies. These studies provide the first preclinical evidence that B7H3.CAR EBVSTs can overcome the challenges of heterogeneous tumor antigen expression and antigen escape, demonstrating potent and durable efficacy against solid tumors expressing a spectrum of B7-H3 levels.

As a result of CD45RA depletion and expansion of EBVSTs in the manufacturing process, the B7H3.CAR EBVST product consists predominantly of effector memory (TEM) or effector memory CD45RA positive (TEMRA) T cell subsets. One potential drawback of our strategy is that our CAR T infusion product contains a low proportion of Stem cell-like memory (TSCM) and central memory (TCM) T cells, which has been demonstrated to correlate with increased cell persistence and improved antitumor responses (46, 47). In addition, we noted that activation induced expression of immune checkpoint markers on B7H3.CAR EBVSTs. To study whether these factors impact B7H3.CAR EBVSTs long-term persistence and activity, we subjected B7H3.CAR EBVST-treated mice to a tumor rechallenge and observed that B7H3.CAR EBVSTs maintained robust activity and persistence by rejecting NCI-N87 tumors.

Leveraging on the observation that the P2A5 VHH clone in our B7H3.CAR recognizes both human and mouse B7-H3, we looked for toxicities in immunocompetent mice bearing syngeneic tumors. Although the P2A5 VHH binding affinity to murine B7-H3 is roughly 15 times weaker than its affinity to human B7-H3, the pronounced potency of murine B7H3.CAR T cells suggests any untoward toxicity should still be detectable in immunocompetent mice. We did not observe any overt signs of toxicity in mice which received B7H3.CAR EBVSTs, apart from minor reductions in T and NK cell populations in the bone marrow and possible chronic pancreatitis, both which would be clinically manageable in humans. While the lack of significant on-target off-tumor toxicity in B7H3.CAR T cells aligns with findings from other groups (48–50) and is reinforced by observations (3) including our own that underscore a lower abundance of B7-H3 in normal tissues compared with tumors, it may be prudent to conduct additional syngeneic safety studies in a larger cohort of mice. When we extended our studies to human immune cells *in vitro*, we observed that while resting monocytes expressed low levels of B7-H3, they upregulated B7-H3 in the presence of GMCSF or allogeneic T cells. This rendered monocytes susceptible to cytolysis by B7H3.CAR EBVSTs under these conditions, while allogeneic or cytokines-stimulated T, B, and NK cells were unaffected because of their lack of B7-H3 expression. Furthermore, we noted that memory T cells, upon reactivation with their cognate antigens, did not upregulate B7-H3 expression, suggesting that they were unlikely to be targets of B7H3.CAR EBVSTs. Our observations in humanized mice also raise the possibility of on-target off-tumor targeting of myeloid cells including monocytes, dendritic cells, and neutrophils. However, it is important to note that this may not necessarily be detrimental, especially in the specific case of monocytes. We observed a low incidence of CRS following B7H3.CAR EBVST treatment in these mice, likely due to B7H3.CAR EBVST depletion of B7-H3–expressing myeloid cells which are key mediators of CRS. This quality of B7H3.CAR EBVSTs may distinguish them from other CAR T-cell therapies which have been linked to significant incidence of CRS, hemophagocytic lymphohistiocytosis/macrophage activation syndrome, and Immune effector Cell-Associated Neurotoxicity Syndrome (51). As the onset of CRS typically manifests and peaks in 2 weeks after CAR T-cell therapy, reductions in the myeloid compartment in patients can be clinically managed through administration of myeloid colony-stimulating factors after this window. We employed CD19.CAR T cells as positive controls because CRS is extensively documented in CD19-positive hematologic malignancies. Nonetheless, we acknowledge that comparing CD19.CAR with B7H3.CAR EBVSTs in a B7-H3–transduced NALM-6 leukemia model would provide a more equitable comparison. We intend to address this in forthcoming studies. Finally, we observed that B7H3.CAR EBVSTs only targeted matured lineages of HSPCs but not resting or early HSPCs, likely a result of B7-H3 upregulation during lineage differentiation. These observations imply that reserves of early progenitor subsets with the capability to replenish various hematopoietic populations remained intact and functional. Our findings align with recent data highlighting the favorable antitumor activity and tolerability of systemic autologous CAR T cells targeting B7-H3 in pediatric patients with solid tumors (52). Certainly, conducting additional safety studies would be essential for a comprehensive evaluation of the safety profile of B7H3.CAR-armored EBVSTs.

While CAR T cells have made significant inroads in the treatment of hematologic malignancies, their success in solid tumor types has been limited, impeded in large part by the immunosuppressive TME populated by MDSCs and tumor-associated macrophages (TAM). Our study reveals that B7H3.CAR EBVSTs effectively target MDSCs and in turn help reverse the T-cell immunosuppressive effects driven by MDSCs. While we have yet to perform similar studies on TAMs, reports of B7-H3 enrichment in TAMs present in human colorectal cancer, NSCLC, and TNBC (53–55) imply that these effects of B7H3.CAR EBVSTs are likely to extend to TAMs. We therefore propose that B7H3.CAR EBVSTs not only directly kill tumor cells, but by targeting inhibitory MDSCs, can also counteract some of the immunosuppressive forces present in the TME.

In summary, we have successfully developed B7H3.CAR EBVSTs as a tumor-agnostic strategy to target B7-H3–positive solid tumors. In addition to a clinically manageable on-target off-tumor safety profile, B7H3.CAR EBVSTs are associated with minimal treatment-induced CRS. We also provide evidence that B7H3.CAR EBVSTs can target MDSCs and reverse their immunosuppressive effects. Despite the daunting challenges that targeting solid tumors present, we believe that B7H3.CAR EBVSTs, with their activity against tumor cells, MDSCs and potentially tumor stroma, are well positioned for clinical application in solid tumors.

## Supplementary Material

Supplementary Data Supplementary Material and Methods

Supplementary figure 1 Supplementary figure 1

Table S1 Supplementary table 1

Supplementary figure 2 Supplementary figure 2

Supplementary figure 3 Supplementary figure 3

Supplementary figure 4 Supplementary figure 4

Supplementary figure 5 Supplementary figure 5

Supplementary figure 6 Supplementary figure 6

Supplementary figure 7 Supplementary figure 7

Supplementary figure 8 Supplementary figure 8

## References

[1] Cappell KM , Kochenderfer JN. Long-term outcomes following CAR T cell therapy: what we know so far. Nat Rev Clin Oncol 2023;20:359–71. 37055515 10.1038/s41571-023-00754-1 PMC10100620

[2] Flugel CL , Majzner RG, Krenciute G, Dotti G, Riddell SR, Wagner DL, . Overcoming on-target, off-tumour toxicity of CAR T cell therapy for solid tumours. Nat Rev Clin Oncol 2023;20:49–62. 36418477 10.1038/s41571-022-00704-3 PMC10278599

[3] Seaman S , Zhu Z, Saha S, Zhang XM, Yang MY, Hilton MB, . Eradication of tumors through simultaneous ablation of CD276/B7-H3-positive tumor cells and tumor vasculature. Cancer Cell 2017;31:501–15. 28399408 10.1016/j.ccell.2017.03.005 PMC5458750

[4] Sun M , Richards S, Prasad DV, Mai XM, Rudensky A, Dong C. Characterization of mouse and human B7-H3 genes. J Immunol 2002;168:6294–7. 12055244 10.4049/jimmunol.168.12.6294

[5] Ye Z , Zheng Z, Li X, Zhu Y, Zhong Z, Peng L, . B7-H3 overexpression predicts poor survival of cancer patients: a meta-analysis. Cell Physiol Biochem 2016;39:1568–80. 27626927 10.1159/000447859

[6] Flem-Karlsen K , Fodstad O, Tan M, Nunes-Xavier CE. B7-H3 in cancer - beyond immune regulation. Trends Cancer 2018;4:401–4. 29860983 10.1016/j.trecan.2018.03.010

[7] Zhou WT , Jin WL. B7-H3/CD276: an emerging cancer immunotherapy. Front Immunol 2021;12:701006. 34349762 10.3389/fimmu.2021.701006 PMC8326801

[8] Jayaraman J , Mellody MP, Hou AJ, Desai RP, Fung AW, Pham AHT, . CAR-T design: elements and their synergistic function. EBioMedicine 2020;58:102931. 32739874 10.1016/j.ebiom.2020.102931 PMC7393540

[9] Fujiwara K , Masutani M, Tachibana M, Okada N. Impact of scFv structure in chimeric antigen receptor on receptor expression efficiency and antigen recognition properties. Biochem Biophys Res Commun 2020;527:350–7. 32216966 10.1016/j.bbrc.2020.03.071

[10] Long AH , Haso WM, Shern JF, Wanhainen KM, Murgai M, Ingaramo M, . 4-1BB costimulation ameliorates T cell exhaustion induced by tonic signaling of chimeric antigen receptors. Nat Med 2015;21:581–90. 25939063 10.1038/nm.3838 PMC4458184

[11] Gil D , Schrum AG. Strategies to stabilize compact folding and minimize aggregation of antibody-based fragments. Adv Biosci Biotechnol 2013;4:73–84. 25635232 10.4236/abb.2013.44A011 PMC4307952

[12] Bao C , Gao Q, Li LL, Han L, Zhang B, Ding Y, . The application of nanobody in CAR-T therapy. Biomolecules 2021;11:238. 33567640 10.3390/biom11020238 PMC7914546

[13] Safarzadeh Kozani P , Naseri A, Mirarefin SMJ, Salem F, Nikbakht M, Evazi Bakhshi S, . Nanobody-based CAR-T cells for cancer immunotherapy. Biomark Res 2022;10:24. 35468841 10.1186/s40364-022-00371-7 PMC9036779

[14] Caldwell KJ , Gottschalk S, Talleur AC. Allogeneic CAR cell therapy-more than a pipe dream. Front Immunol 2020;11:618427. 33488631 10.3389/fimmu.2020.618427 PMC7821739

[15] Depil S , Duchateau P, Grupp SA, Mufti G, Poirot L. 'Off-the-shelf' allogeneic CAR T cells: development and challenges. Nat Rev Drug Discov 2020;19:185–99. 31900462 10.1038/s41573-019-0051-2

[16] Benjamin R , Graham C, Yallop D, Jozwik A, Mirci-Danicar OC, Lucchini G, . Genome-edited, donor-derived allogeneic anti-CD19 chimeric antigen receptor T cells in paediatric and adult B-cell acute lymphoblastic leukaemia: results of two phase 1 studies. Lancet 2020;396:1885–94. 33308471 10.1016/S0140-6736(20)32334-5 PMC11773457

[17] Hu Y , Zhou Y, Zhang M, Zhao H, Wei G, Ge W, . Genetically modified CD7-targeting allogeneic CAR-T cell therapy with enhanced efficacy for relapsed/refractory CD7-positive hematological malignancies: a phase I clinical study. Cell Res 2022;32:995–1007. 36151216 10.1038/s41422-022-00721-y PMC9652391

[18] Mailankody S , Matous JV, Chhabra S, Liedtke M, Sidana S, Oluwole OO, . Allogeneic BCMA-targeting CAR T cells in relapsed/refractory multiple myeloma: phase 1 UNIVERSAL trial interim results. Nat Med 2023;29:422–9. 36690811 10.1038/s41591-022-02182-7

[19] Enache OM , Rendo V, Abdusamad M, Lam D, Davison D, Pal S, . Cas9 activates the p53 pathway and selects for p53-inactivating mutations. Nat Genet 2020;52:662–8. 32424350 10.1038/s41588-020-0623-4 PMC7343612

[20] Nahmad AD , Reuveni E, Goldschmidt E, Tenne T, Liberman M, Horovitz-Fried M, . Frequent aneuploidy in primary human T cells after CRISPR-Cas9 cleavage. Nat Biotechnol 2022;40:1807–13. 35773341 10.1038/s41587-022-01377-0 PMC7613940

[21] Prockop S , Doubrovina E, Suser S, Heller G, Barker J, Dahi P, . Off-the-shelf EBV-specific T cell immunotherapy for rituximab-refractory EBV-associated lymphoma following transplantation. J Clin Invest 2020;130:733–47. 31689242 10.1172/JCI121127 PMC6994129

[22] Kochenderfer JN , Yu Z, Frasheri D, Restifo NP, Rosenberg SA. Adoptive transfer of syngeneic T cells transduced with a chimeric antigen receptor that recognizes murine CD19 can eradicate lymphoma and normal B cells. Blood 2010;116:3875–86. 20631379 10.1182/blood-2010-01-265041 PMC2981541

[23] Sharma S , Woods M, Mehta NU, Sauer T, Parikh KS, Schmuck-Henneresse M, . Naive T cells inhibit the outgrowth of intractable antigen-activated memory T cells: implications for T-cell immunotherapy. J Immunother Cancer 2023;11:e006267. 37072346 10.1136/jitc-2022-006267 PMC10124261

[24] Parihar R , Rivas C, Huynh M, Omer B, Lapteva N, Metelitsa LS, . NK cells expressing a chimeric activating receptor eliminate MDSCs and rescue impaired CAR-T cell activity against solid tumors. Cancer Immunol Res 2019;7:363–75. 30651290 10.1158/2326-6066.CIR-18-0572 PMC7906796

[25] Mao R , Kong W, He Y. The affinity of antigen-binding domain on the antitumor efficacy of CAR T cells: moderate is better. Front Immunol 2022;13:1032403. 36325345 10.3389/fimmu.2022.1032403 PMC9618871

[26] Xiao L , Guan X, Xiang M, Wang Q, Long Q, Yue C, . B7 family protein glycosylation: promising novel targets in tumor treatment. Front Immunol 2022;13:1088560. 36561746 10.3389/fimmu.2022.1088560 PMC9763287

[27] Khan M , Arooj S, Wang H. Soluble B7-CD28 family inhibitory immune checkpoint proteins and anti-cancer immunotherapy. Front Immunol 2021;12:651634. 34531847 10.3389/fimmu.2021.651634 PMC8438243

[28] Chapoval AI , Ni J, Lau JS, Wilcox RA, Flies DB, Liu D, . B7-H3: a costimulatory molecule for T cell activation and IFN-gamma production. Nat Immunol 2001;2:269–74. 11224528 10.1038/85339

[29] Hombach AA , Gorgens A, Chmielewski M, Murke F, Kimpel J, Giebel B, . Superior therapeutic index in lymphoma therapy: CD30(+) CD34(+) hematopoietic stem cells resist a chimeric antigen receptor T-cell attack. Mol Ther 2016;24:1423–34. 27112062 10.1038/mt.2016.82 PMC5023391

[30] Norelli M , Camisa B, Barbiera G, Falcone L, Purevdorj A, Genua M, . Monocyte-derived IL-1 and IL-6 are differentially required for cytokine-release syndrome and neurotoxicity due to CAR T cells. Nat Med 2018;24:739–48. 29808007 10.1038/s41591-018-0036-4

[31] Dunmire SK , Verghese PS, Balfour HH Jr. Primary Epstein-Barr virus infection. J Clin Virol 2018;102:84–92. 29525635 10.1016/j.jcv.2018.03.001

[32] Amyes E , Hatton C, Montamat-Sicotte D, Gudgeon N, Rickinson AB, McMichael AJ, . Characterization of the CD4+ T cell response to Epstein-Barr virus during primary and persistent infection. J Exp Med 2003;198:903–11. 12975456 10.1084/jem.20022058 PMC2194204

[33] Appay V , Dunbar PR, Callan M, Klenerman P, Gillespie GM, Papagno L, . Memory CD8+ T cells vary in differentiation phenotype in different persistent virus infections. Nat Med 2002;8:379–85. 11927944 10.1038/nm0402-379

[34] Melenhorst JJ , Castillo P, Hanley PJ, Keller MD, Krance RA, Margolin J, . Graft versus leukemia response without graft-versus-host disease elicited by adoptively transferred multivirus-specific T-cells. Mol Ther 2015;23:179–83. 25266309 10.1038/mt.2014.192 PMC4426803

[35] Quach DH , Lulla P, Rooney CM. Banking on virus-specific T cells to fulfill the need for off-the-shelf cell therapies. Blood 2023;141:877–85. 36574622 10.1182/blood.2022016202 PMC10023738

[36] Quach DH , Ramos CA, Lulla PD, Sharma S, Ganesh HR, Nouraee N, . CD30.CAR-modified epstein-barr virus-specific T cells (CD30.CAR EBVSTs) provide a safe and effective off-the-shelf therapy for patients with CD30-positive lymphoma. Blood 2022;140:412–4.

[37] Ramos CA , Quach DH, Lulla PD, Rouce RH, Sharma S, Ganesh HR, . Off-the-shelf CD30.CAR-modified Epstein-Barr virus-specific T cells (CD30.CAR EBVSTS) provide a safe and effective therapy for patients with Hodgkin lymphoma (HL). Hematol Oncol 2023;41:83–5.

[38] Caushi JX , Zhang J, Ji Z, Vaghasia A, Zhang B, Hsiue EH, . Transcriptional programs of neoantigen-specific TIL in anti-PD-1-treated lung cancers. Nature 2021;596:126–32. 34290408 10.1038/s41586-021-03752-4 PMC8338555

[39] Scheper W , Kelderman S, Fanchi LF, Linnemann C, Bendle G, de Rooij MAJ, . Low and variable tumor reactivity of the intratumoral TCR repertoire in human cancers. Nat Med 2019;25:89–94. 30510250 10.1038/s41591-018-0266-5

[40] Simoni Y , Becht E, Fehlings M, Loh CY, Koo SL, Teng KWW, . Bystander CD8(+) T cells are abundant and phenotypically distinct in human tumour infiltrates. Nature 2018;557:575–9. 29769722 10.1038/s41586-018-0130-2

[41] Lapteva N , Gilbert M, Diaconu I, Rollins LA, Al-Sabbagh M, Naik S, . T-cell receptor stimulation enhances the expansion and function of CD19 chimeric antigen receptor-expressing T cells. Clin Cancer Res 2019;25:7340–50. 31558475 10.1158/1078-0432.CCR-18-3199 PMC7062259

[42] Tanaka M , Tashiro H, Omer B, Lapteva N, Ando J, Ngo M, . Vaccination targeting native receptors to enhance the function and proliferation of chimeric antigen receptor (CAR)-modified T cells. Clin Cancer Res 2017;23:3499–509. 28183713 10.1158/1078-0432.CCR-16-2138 PMC5511585

[43] Dasari V , McNeil LK, Beckett K, Solomon M, Ambalathingal G, Thuy TL, . Lymph node targeted multi-epitope subunit vaccine promotes effective immunity to EBV in HLA-expressing mice. Nat Commun 2023;14:4371. 37553346 10.1038/s41467-023-39770-1 PMC10409721

[44] Wagner J , Wickman E, DeRenzo C, Gottschalk S. CAR T cell therapy for solid tumors: bright future or dark reality? Mol Ther 2020;28:2320–39. 32979309 10.1016/j.ymthe.2020.09.015 PMC7647674

[45] Hou AJ , Chen LC, Chen YY. Navigating CAR-T cells through the solid-tumour microenvironment. Nat Rev Drug Discov 2021;20:531–50. 33972771 10.1038/s41573-021-00189-2

[46] Biasco L , Izotova N, Rivat C, Ghorashian S, Richardson R, Guvenel A, . Clonal expansion of T memory stem cells determines early anti-leukemic responses and long-term CAR T cell persistence in patients. Nat Cancer 2021;2:629–42. 34345830 10.1038/s43018-021-00207-7 PMC7611448

[47] Xu Y , Zhang M, Ramos CA, Durett A, Liu E, Dakhova O, . Closely related T-memory stem cells correlate with *in vivo* expansion of CAR.CD19-T cells and are preserved by IL-7 and IL-15. Blood 2014;123:3750–9. 24782509 10.1182/blood-2014-01-552174 PMC4055922

[48] Du H , Hirabayashi K, Ahn S, Kren NP, Montgomery SA, Wang X, . Antitumor responses in the absence of toxicity in solid tumors by targeting B7-H3 via chimeric antigen receptor T cells. Cancer Cell 2019;35:221–37. 30753824 10.1016/j.ccell.2019.01.002 PMC6645919

[49] Haydar D , Houke H, Chiang J, Yi Z, Ode Z, Caldwell K, . Cell-surface antigen profiling of pediatric brain tumors: B7-H3 is consistently expressed and can be targeted via local or systemic CAR T-cell delivery. Neuro Oncol 2021;23:999–1011. 33320196 10.1093/neuonc/noaa278 PMC8168826

[50] Lichtman EI , Du H, Shou P, Song F, Suzuki K, Ahn S, . Preclinical evaluation of B7-H3-specific chimeric antigen receptor T cells for the treatment of acute myeloid leukemia. Clin Cancer Res 2021;27:3141–53. 33531429 10.1158/1078-0432.CCR-20-2540 PMC8248479

[51] Sheth VS , Gauthier J. Taming the beast: CRS and ICANS after CAR T-cell therapy for ALL. Bone Marrow Transplant 2021;56:552–66. 33230186 10.1038/s41409-020-01134-4 PMC8592274

[52] Pinto NR , Albert CM, Taylor M, Wilson A, Rawlings-Rhea S, Huang W, . STRIVE-02: a first-in-human phase 1 trial of systemic B7H3 CAR T cells for children and young adults with relapsed/refractory solid tumors. J Clin Oncol 2022;40:16s, (suppl; abstr 10011).

[53] Chen C , Shen Y, Qu QX, Chen XQ, Zhang XG, Huang JA. Induced expression of B7-H3 on the lung cancer cells and macrophages suppresses T-cell mediating anti-tumor immune response. Exp Cell Res 2013;319:96–102. 22999863 10.1016/j.yexcr.2012.09.006

[54] Cheng N , Bei Y, Song Y, Zhang W, Xu L, Zhang W, . B7-H3 augments the pro-angiogenic function of tumor-associated macrophages and acts as a novel adjuvant target for triple-negative breast cancer therapy. Biochem Pharmacol 2021;183:114298. 33153969 10.1016/j.bcp.2020.114298

[55] Mao Y , Chen L, Wang F, Zhu D, Ge X, Hua D, . Cancer cell-expressed B7-H3 regulates the differentiation of tumor-associated macrophages in human colorectal carcinoma. Oncol Lett 2017;14:6177–83. 29113264 10.3892/ol.2017.6935 PMC5661406

