# Supplementary material for: B7-H3–Targeting Chimeric Antigen Receptors Epstein-Barr Virus–specific T Cells Provides a Tumor Agnostic Off-The-Shelf Therapy Against B7-H3–positive Solid Tumors
Source: Cancer Res Commun. 2024 Jun 4;4(6):1410–29. doi: 10.1158/2767-9764.CRC-23-0538 (PMC11149603; doi:10.1158/2767-9764.CRC-23-0538)
Supplement: Supplementary Data — Supplementary Material and Methods [file crc-23-0538-s01.docx]

**Supplementary Materials and Methods**

**Generation of B7-H3 targeting human and murine CAR T cells**

For generation of human CAR T cells, human PBMCs were stimulated with plate-coated anti-CD3/CD28 and cultured in cytotoxic T lymphocyte (CTL) medium to generate activated T cells (ATCs). For generation of human CAR EBVSTs, EBVSTs were first generated by pulsing CD45RA-depleted human PBMCs with pooled EBV pepmixes and maintained in virus specific T cell (VST) medium. Both CTL and VST culture media were supplemented with 10 ng/ml IL-7 and IL-15 (R&D Systems). On days 3 and 5 post-activation, ATCs and EBVSTs were transduced with retrovirus bearing the B7H3.CAR transgene or left untransduced. Untransduced (UT) or B7H3.CAR EBVSTs were re-stimulated on day 9 with irradiated feeder cells. On day 11 post-transduction, T cell products were frozen down using CryoStor® and stored in liquid nitrogen.

**Real-time Cytotoxicity and Serial Killing Potency Assays**

Tumor target cells were added onto E-plates following manufacturer’s manual (Agilent Technologies).

For cytotoxicity studies, human or murine effector CAR-T cells were plated onto the E-plates at various CAR-T: target cell ratios and cytotoxicity was monitored for 48 hours. In some experiments, 50μl of culture supernatant was collected and replenished after 24 hours for Interferon-γ ELISA using the Human IFN-gamma Quantikine ELISA Kit (R&D Systems).

In serial killing assays, CAR-T cells were plated onto the E-plates at a defined CAR-T: target cell ratio of 1:2. The E-plates were returned to the xCELLigence® system and cytotoxicity was monitored for 48 hours. After 48 hours, the suspensions of CAR-T cells were harvested, counted and added to plates of target cells seeded 24 hours earlier to set up Encounter 2. This was repeated for 5 encounters. After each encounter, 1 set of cells was stained with viability dyes and antibodies against camelid VHH, CD3, CD4, CD8, B7-H3, PD-1, Tim3 and LAG3 with addition of counting beads to enumerate the effector cell populations present after each round of target encounter.

Cytolysis was calculated as (cell count in target-only control well – cell count in assay well) / cell count in target-only control well.

**Cell Culture Media Components**

| **Medium Name** | **Components** | **Composition** | **Supplier** |
| --- | --- | --- | --- |
| Cytotoxic T Lymphocyte (CTL) Medium | Advanced RPMI 1640 Medium | 45% | Thermo-Fisher Scientific |
|  | Click’s Medium | 45% | FUJIFILM Irvine Scientific) |
|  | Fetal Bovine Serum | 10% | Thermo-Fisher Scientific |
|  | GlutaMAX™ Supplement | 1X | Thermo-Fisher Scientific |
| Virus Specific T Cell (VST) Medium | Advanced RPMI 1640 Medium | 47.5% | Thermo-Fisher Scientific |
|  | Click’s Medium | 47.5% | FUJIFILM Irvine Scientific) |
|  | Human Platelet Lysate | 5% | Sexton Biotechnologies |
|  | GlutaMAX™ Supplement | 2mM | Thermo-Fisher Scientific |
| Mouse T Cell Medium | RPMI 1640 Medium | 90% | Thermo-Fisher Scientific |
|  | Fetal Bovine Serum | 10% | Thermo-Fisher Scientific |
|  | HEPES | 20nM | Thermo-Fisher Scientific |
|  | Sodium pyruvate | 1mM | Thermo-Fisher Scientific |
|  | MEM NEAA | 100μM | Thermo-Fisher Scientific |
|  | Beta-mercaptoethanol | 1x | Thermo-Fisher Scientific |
| Cell Line Media (for all cell lines except SW480, HT-29, MDA-MB-231, MDA-MB-468 and MKN-45) | RPMI 1640 Medium | 90% | Thermo-Fisher Scientific |
|  | Fetal Bovine Serum | 10% | Thermo-Fisher Scientific |
|  | Penicillin-Streptomycin (10000 U/mL) | 1x | Thermo-Fisher Scientific |
| Cell Line Media (for SW480, MDA-MB-231, MDA-MB-468 cells) | Leibovitz's L-15 Medium |  | Thermo-Fisher Scientific |
|  | Fetal Bovine Serum | 10% | Thermo-Fisher Scientific |
|  | Penicillin-Streptomycin (10000 U/mL) | 1x | Thermo-Fisher Scientific |
| Cell Line Media (for HT-29 cells) | McCoy's 5a Medium Modified |  | Thermo-Fisher Scientific |
|  | Fetal Bovine Serum | 10% | Thermo-Fisher Scientific |
|  | Penicillin-Streptomycin (10000 U/mL) | 1x | Thermo-Fisher Scientific |
| Cell Line Media (for MKN-45 cells) | RPMI 1640 Medium | 80% | Thermo-Fisher Scientific |
|  | Fetal Bovine Serum | 20% | Thermo-Fisher Scientific |
|  | Penicillin-Streptomycin (10000 U/mL) | 1x | Thermo-Fisher Scientific |
| Cell Line Media (for RD114 packaging cells) | DMEM (without L-glutamine) High glucose | 90% | Thermo-Fisher Scientific |
|  | Fetal Bovine Serum | 10% | Thermo-Fisher Scientific |
|  | GlutaMAX™ Supplement | 1x | Thermo-Fisher Scientific |
| MACS Buffer | Phosphate Buffered Saline, pH7.4 | 100% | Thermo-Fisher Scientific |
|  | Bovine Serum Albumin | 0.5% | Sigma-Aldrich |
|  | EDTA | 2mM | Thermo-Fisher Scientific |

**Flow Cytometry Antibodies/ Reagents Table**

| **Antibodies/ Reagents** | **Fluorochrome** | **Clone** | **Supplier** | **RRID** |
| --- | --- | --- | --- | --- |
| Live/Dead™ NIR viability dye | NA | NA | Thermo Fisher | NA |
| Live/Dead™ AQUA viability dye | NA | NA | Thermo Fisher | NA |
| Live/Dead™ UV viability dye | NA | NA | Thermo Fisher | NA |
| CountBright™ Absolute Counting Beads | NA | NA | Invitrogen | NA |
| Human CD45 | BUV395 | H130 | BD Biosciences | AB_2869519 |
| Human CD3 | BUV395 | SK7 | BD Biosciences | AB_2744382 |
| Human CD3 | BB700 | SK7 | BD Biosciences | AB_2860004 |
| Human CD3 | PE-Cy7 | SK7 | BD Biosciences | AB_396896 |
| Human CD3 | BUV563 | SK7 | BD Biosciences | AB_2870922 |
| Human CD3 | BV510 | OKT3 | Biolegend | AB_2561943 |
| Human CD3 | BV650 | SK7 | BD Biosciences | AB_2738535 |
| Human CD3 (NA/LE purified) | NA | OKT3 | BD Biosciences | AB_2869821 |
| Human CD4 | BUV805 | SK3 | BD Biosciences | AB_2870177 |
| Human CD28 (purified) |  | CD28.2 | BD Biosciences | AB_396492 |
| Human CD8a | BV650 | SK1 | BioLegend | AB_2564510 |
| Human CD8a | BV605 | RPA-T8 | BD Biosciences | AB_2740141 |
| Human CD8a | BUV563 | RPA-T8 | BD Biosciences | AB_2870200 |
| Human CD56 | AF700 | B159 | BioLegend | AB_604104 |
| Human CD56 | BV650 | B159 | BioLegend | AB_2563838 |
| Human CD56 | BUV563 | B159 | BD Biosciences | AB_2870874 |
| Human CD19 | APC-Cy7 | SJ25C1 | BD Biosciences | AB_396873 |
| Human CD19 | PE | SJ25C1 | BD Biosciences | AB_400018 |
| Human CD19 | PE-Cy5 | HIB19 | BD Biosciences | AB_395814 |
| Human B7-H3 | BB700 | 7-517 | BD Biosciences | AB_2743277 |
| Human B7-H3 | PE | MIH42 | BD Biosciences | AB_10720987 |
| Human CD45RA | BUV615 | HI100 | BD Biosciences | AB_2875550 |
| Human CD45RA | BV711 | HI100 | BioLegend | AB_2563815 |
| Human CCR7 | BV650 | G043H7 | BioLegend | AB_2563867 |
| Human CCR7 | BV650 | 150503 | BD Biosciences | AB_2869851 |
| Human PD-1 | BV785 | EH12.1 | BioLegend | AB_2563443 |
| Human TIM-3 | BUV615 | 7D3 | BD Biosciences | AB_2875880 |
| Human LAG3 | BV421 | 11C3C65 | BioLegend | AB_2629797 |
| Human LAG3 | BV711 | 11C3C65 | BioLegend | AB_2716125 |
| Human IFN-γ | PE | B27 | BioLegend | AB_315440 |
| Human TNF-α | APC | Mab11 | BD Biosciences | AB_398566 |
| Human CD25 | BUV395 | 2A3 | BD Biosciences | AB_2738556 |
| Human CD14 | APC-Cy7 | M5E2 | BioLegend | AB_493695 |
| Human CD14 | BUV805 | M5E2 | BD Biosciences | AB_2870189 |
| Human CD15 | BUV661 | W6D3 | BD Biosciences | AB_2871054 |
| Human CD16 | BUV737 | 3G8 | BD Biosciences | Not found |
| Human CD33 | BV421 | HIM3-4 | BD Biosciences | AB_2742169 |
| Human CD34 | APC | 561 | BioLegend | AB_2228972 |
| Human CD133 | PE | 7 | BioLegend | AB_2632880 |
| Human CD38 | BV421 | HIT2 | BD Biosciences | AB_11151894 |
| Human CD10 | PE-Cy7 | HI10a | BD Biosciences | AB_2739153 |
| Human CD11b | BV605 | ICRF44 | BioLegend | AB_2562021 |
| Human CD11c | PE-Cy7 | B-ly6 | BD Biosciences | AB_10611859 |
| Human CD66b | AF700 | 6/40c | Biolegend | AB_2566038 |
| Human CD68 | BV786 | FA-11 | Thermo-Fisher | Not found |
| Human HLA-DR | PE-Cy5 | G46-6 | BD Biosciences | AB_396147 |
| Human IL-10 | BV711 | JES3-9D7 | BD Biosciences | AB_2738564 |
| Human TGF-β1 | PE | S200006A | Biolegend | AB_2894444 |
| Human iNos | PE-CF594 | CXNFT | Thermo-Fisher Scientific | Not found |
| Human EPCAM | PE-CF594 | EBA-1 | BD Biosciences | AB_2739219 |
| Human HLA-A3 |  | GAP.A3 | BD Biosciences |  |
| Human CD49d (purified) | NA | L25 | BD Biosciences | AB_400198 |
| Anti-FMC63 Antibody | Biotin | Y45 | Acro Biosystems | Not found |
| Mouse CD45 | APC-Cy7 | 30-F11 | BD Biosciences | AB_396774 |
| Mouse CD45 | BV510 | 30-F11 | BD Biosciences | AB_2734134 |
| Mouse CD45.1 | BV805 | A20 | BD Biosciences | AB_2871266 |
| Mouse CD45.2 | BV786 | 104 | BD Biosciences | AB_2738375 |
| Mouse CD11b | BUV737 | M1/70 | BD Biosciences | AB_2738811 |
| Mouse CD11c | PCP-Cy5.5 | N418 | BD Biosciences | AB_1727422 |
| Mouse Gr-1 | BV711 | RB6-8C5 | BD Biosciences | AB_2740347 |
| Mouse CD19 | BUV805 | ID3 | BD Biosciences | AB_2873424 |
| Mouse NK1.1 | BUV615 | PK136 | BD Biosciences | AB_2875140 |
| Mouse CD49b | APC-Fire-750 | DX5 | Biolegend | AB_2876423 |
| Mouse CD3 | BUV395 | 145-2C11 | BD Biosciences | AB_2738278 |
| Mouse CD4 | BUV395 | RM4-5 | BD Biosciences | AB_2734761 |
| Mouse CD8 | BV650 | 53-6.7 | BD Biosciences | AB_2738084 |
| Mouse F4/80 | R718 | T45-2342 | BD Biosciences | Not found |
| Mouse CD25 | PE-Cy7 | 3C7 | Biolegend | AB_2616762 |
| Mouse PD-1 | PE | RMP1-14 | Biolegend | AB_2566726 |
| Mouse TIM-3 | BV421 | B8.2C12 | Biolegend | AB_2814028 |
| Mouse LAG3 | BV605 | C9B7W | Biolegend | AB_2687209 |
| Human / Mouse B7-H3 |  | EPNCIR122 | Abcam | Not found |
| anti-Camelid VHH | AF647 | 96A3F5 | Genscript | Not found |
| anti-Camelid VHH | FITC | 96A3F5 | Genscript | Not found |
| Streptavin | BV421 | NA | BD Biosciences | AB_2869475 |
| CellTrace^TM^ Violet | NA | NA | Thermo-Fisher Scientific | NA |
| PKH26 Red Fluorescent Cell Linker | NA | NA | Sigma-Aldrich | NA |
| PKH67 Green Fluorescent Cell Linker | NA | NA | Sigma-Aldrich | NA |
| GolgiSTOP and GolgiPlug | NA | NA | BD Biosciences | NA |
| Cytofix/Cytoperm kit | NA | NA | BD Biosciences | NA |

**Immunohistology Antibodies/ Reagents Table**

| **Antibodies** | **Clone** | **Supplier** |
| --- | --- | --- |
| Rabbit Anti-Human B7-H3 mAb | D9M2L | Cell Signalling |
| Rabbit Anti-Human CD3 | Polyclonal | Agilent Technologies |
| Anti-rabbit Poly-HRP-IgG | Polyclonal | Leica Microsystems |
