# Supplementary material for: B7-H3–Targeting Chimeric Antigen Receptors Epstein-Barr Virus–specific T Cells Provides a Tumor Agnostic Off-The-Shelf Therapy Against B7-H3–positive Solid Tumors
Source: Cancer Res Commun. 2024 Jun 4;4(6):1410–29. doi: 10.1158/2767-9764.CRC-23-0538 (PMC11149603; doi:10.1158/2767-9764.CRC-23-0538)
Supplement: Supplementary figure 1 [file crc-23-0538-s02.pdf]

Supplementary figure 1

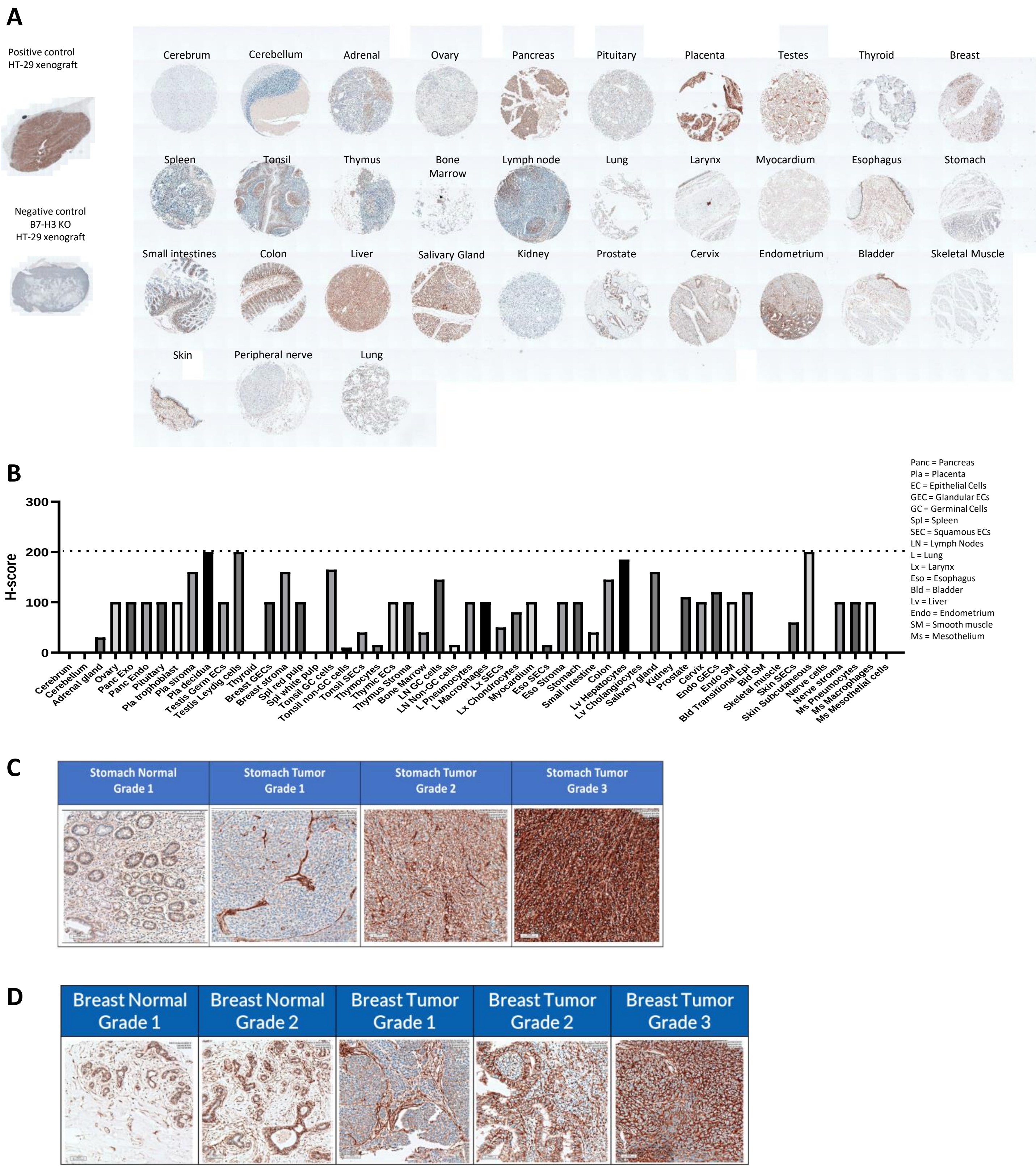

Supplementary figure 1. **A,** B7-H3 expression in IHC staining of human healthy tissues. **B,** H-score for healthy human tissues. Dotted line represents H-score cut-off of 200. **C,** Representative images depicting different levels of B7-H3 expression in IHC staining of gastric cancer samples. **D,** Representative images depicting different levels of B7-H3 expression in IHC staining of triple negative breast cancer samples.
