# Supplementary material for: B7-H3–Targeting Chimeric Antigen Receptors Epstein-Barr Virus–specific T Cells Provides a Tumor Agnostic Off-The-Shelf Therapy Against B7-H3–positive Solid Tumors
Source: Cancer Res Commun. 2024 Jun 4;4(6):1410–29. doi: 10.1158/2767-9764.CRC-23-0538 (PMC11149603; doi:10.1158/2767-9764.CRC-23-0538)
Supplement: Table S1 — Supplementary table 1 [file crc-23-0538-s03.pdf]

Supplementary Table 1

Anatomic Site	B7-H3 staining pattern and distribution
Cerebrum	Negative staining
Cerebellum	Negative staining
Pituitary	Mostly very weak cytoplasmic staining (1+) in epithelial cells.
Thyroid	Negative staining
Lung	Weak cytoplasmic staining (1+) of pneumocytes. Macrophages show prominent membrane staining (1+).
Kidney	Negative staining
Skeletal muscle	Negative staining
Adrenal gland	Weak membrane and cytoplasmic staining (1+) in about 30% of epithelial cells.
Ovary	No germinal epithelium. Very weak staining (1+) in stroma and histiocytes looking cells.
Pancreas	Mostly weak cytoplasmic staining (1+) in epithelial cells. Very weak staining in stroma.
Testis	Mostly weak cytoplasmic staining (1+) in germinal epithelial cells (within seminiferous tubules). Leydig cells show moderate staining (2+).
Spleen	Very weak and uniform staining (1+in red pulp. No definitive staining in white pulp.
Thymus	Weak staining (1+) in islands of thymocytes and stromal cells.
Bone marrow	Weak cytoplasmic staining (1+) of haematopoietic cells.
Larynx	Weak staining (1+) in basal layer of squamous lining and perinuclear area of some chondrocytes. Nonspecific staining in the matrix of the cartilage.
Myocardium	Very weak cytoplasmic staining (1+).
Esophagus	Weak staining (1+) in basal layer of squamous lining. Smooth muscles, blood vessels and stroma show weak staining (1+).
Stomach	Weak staining (1+) in mucosa epithelial cells.
Small intestine	Luminal glands are negative. Submucosal glands show weak staining (1+), fibrovascular cores of glands have stronger staining.
Colon	Weak staining (1+) in mucosa epithelial cells, stroma and plasma cells.
Prostate	Weak to moderate staining (1-2+) in epithelial cells. No staining in stroma.
Cervix	Weak staining (1+) in epithelial cells. Very weak staining of stroma.
Bladder	Weak staining (1+) in transitional epithelium. Bladder muscle is negative.
Peripheral nerve	Very weak staining (1+) in stroma and blood vessels.
Mesothelium	Weak cytoplasmic staining (1+) of pneumocytes and macrophages. Mesothelial cells are negative.
Breast	Ductal epithelial cells showing weak staining (1+). Stroma show weak to moderate staining (1-2+).
Tonsil	Germinal center cells of lymphoid follicles show moderate staining (2+). Basal cells of squamous epithelium and sinusoids show weak staining (1+).
Lymph node	Germinal center cells of lymphoid follicles show weak to moderate staining (1-2+). Weak cytoplasmic staining (1+) of some lymphoid cells.
Liver	Weak to moderate staining (1-2+) in hepatocytes.
Salivary gland	Weak to moderate staining (1-2+) in glandular epithelial cells.
Endometrium	Weak to moderate staining (1-2+) in endometrium and weak staining (1+) in myometrium.
Placenta	Syncitio and cytotrophoblasts show weak staining (1+) whereas stroma inside the chorionic villi show moderate staining (2+). Those looking like decidua cells show moderate to strong staining (2-3+).
Skin/Thigh	Moderate staining (2+) in basal layer of skin and subcutaneous tissue.

Supplementary Table 1. Detailed description of B7-H3 expression and localization in healthy human tissues.
