# Supplementary material for: B7-H3–Targeting Chimeric Antigen Receptors Epstein-Barr Virus–specific T Cells Provides a Tumor Agnostic Off-The-Shelf Therapy Against B7-H3–positive Solid Tumors
Source: Cancer Res Commun. 2024 Jun 4;4(6):1410–29. doi: 10.1158/2767-9764.CRC-23-0538 (PMC11149603; doi:10.1158/2767-9764.CRC-23-0538)
Supplement: Supplementary figure 2 [file crc-23-0538-s04.pdf]

Supplementary figure 2

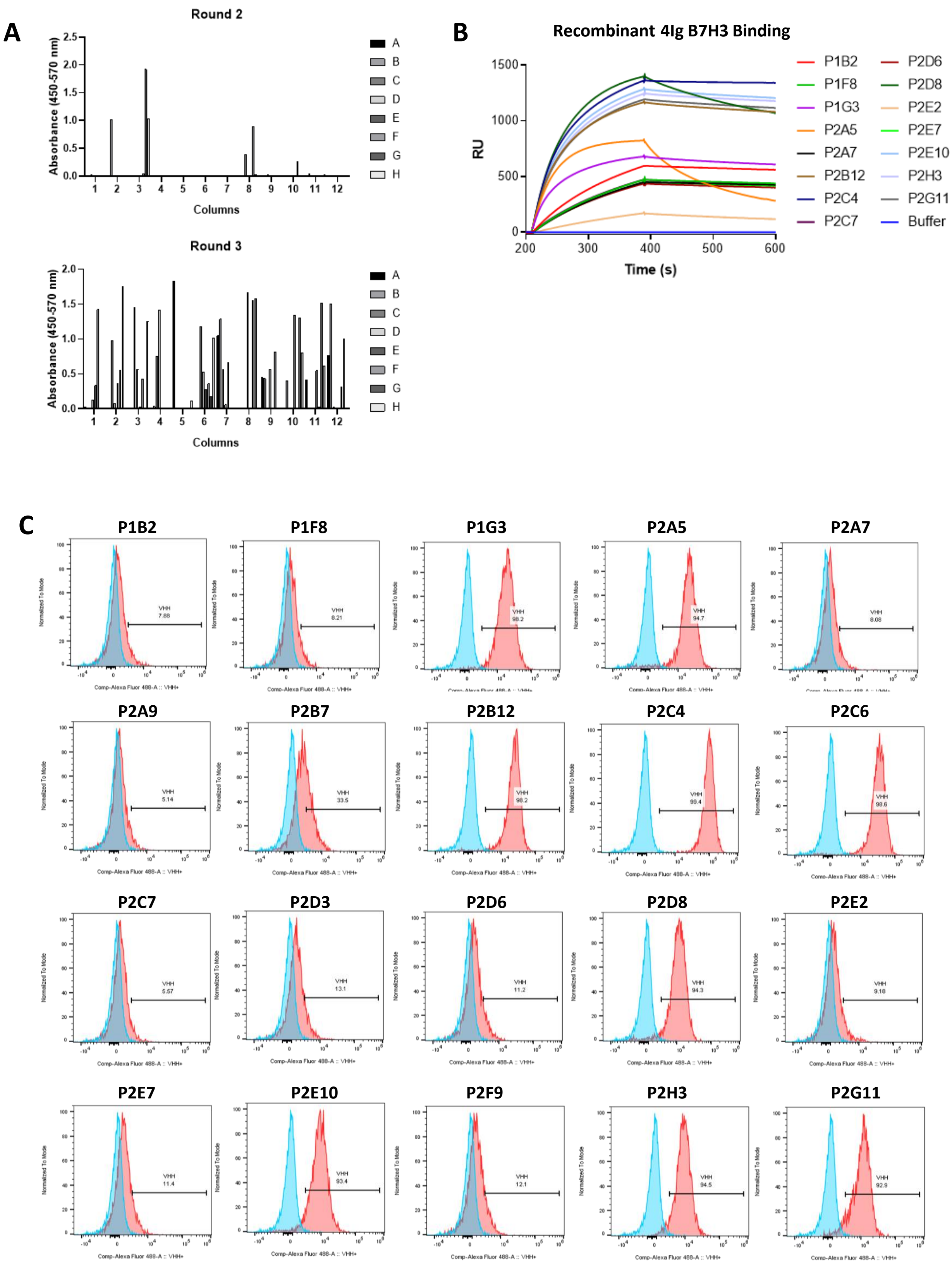

Supplementary figure 2. Identification and characterization of B7-H3-binding VHH from a phage displayed immunized llama library. **A,** B7-H3 binding screened by ELISA of eluted phages from round 2 (top) and round 3 (bottom) of biopanning with recombinant 4-Ig B7-H3. **B,** Surface plasmon resonance screening of B7-H3 binding to purified lead VHH candidates. **C,** Flow cytometry screening for binding of lead VHH candidates to B7-H3 expressing HepG2 cells. Histograms represent isotype stain in blue and VHH binding in red.
