# Supplementary material for: B7-H3–Targeting Chimeric Antigen Receptors Epstein-Barr Virus–specific T Cells Provides a Tumor Agnostic Off-The-Shelf Therapy Against B7-H3–positive Solid Tumors
Source: Cancer Res Commun. 2024 Jun 4;4(6):1410–29. doi: 10.1158/2767-9764.CRC-23-0538 (PMC11149603; doi:10.1158/2767-9764.CRC-23-0538)
Supplement: Supplementary figure 3 [file crc-23-0538-s05.pdf]

Supplementary figure 3

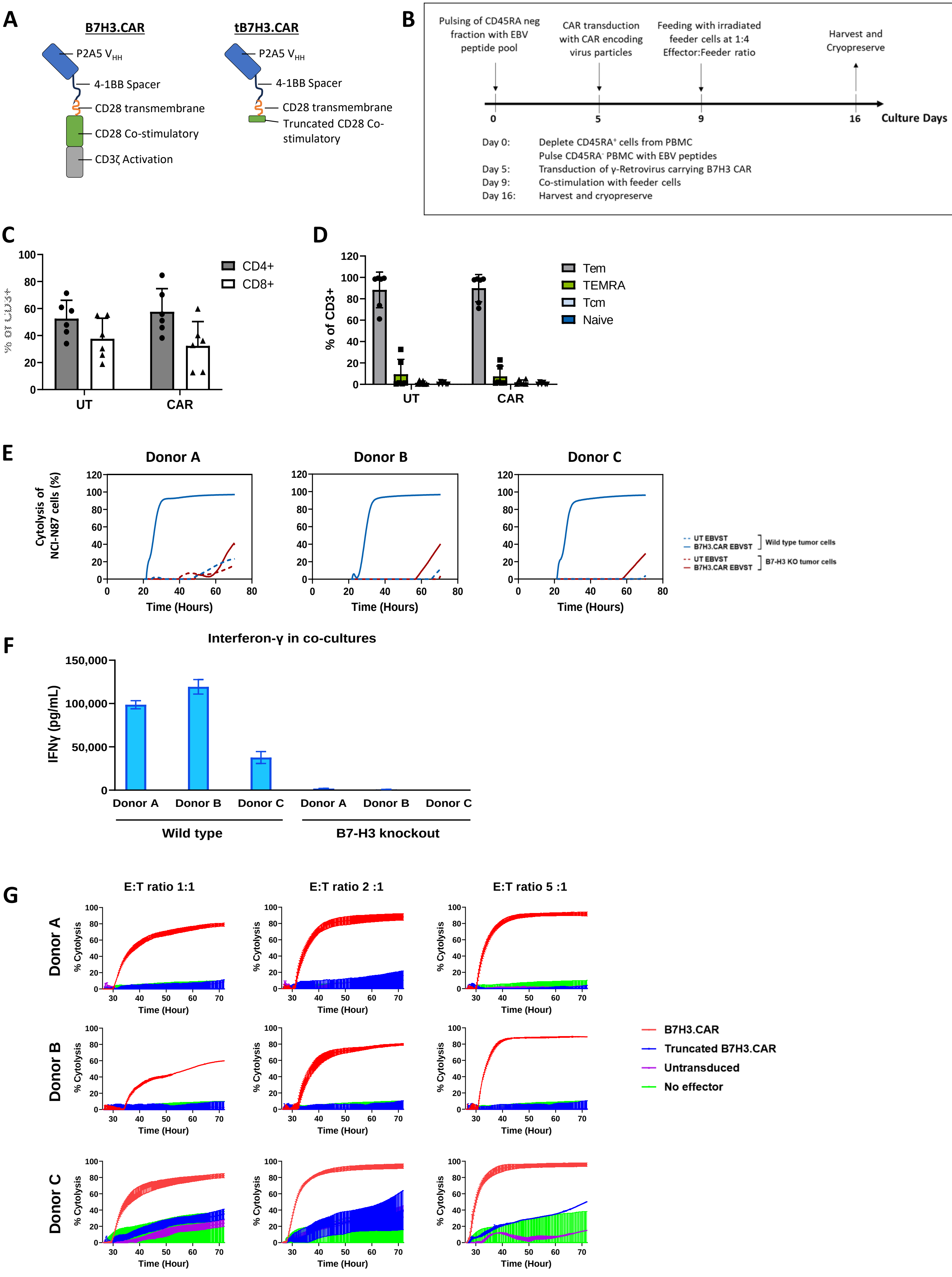

H

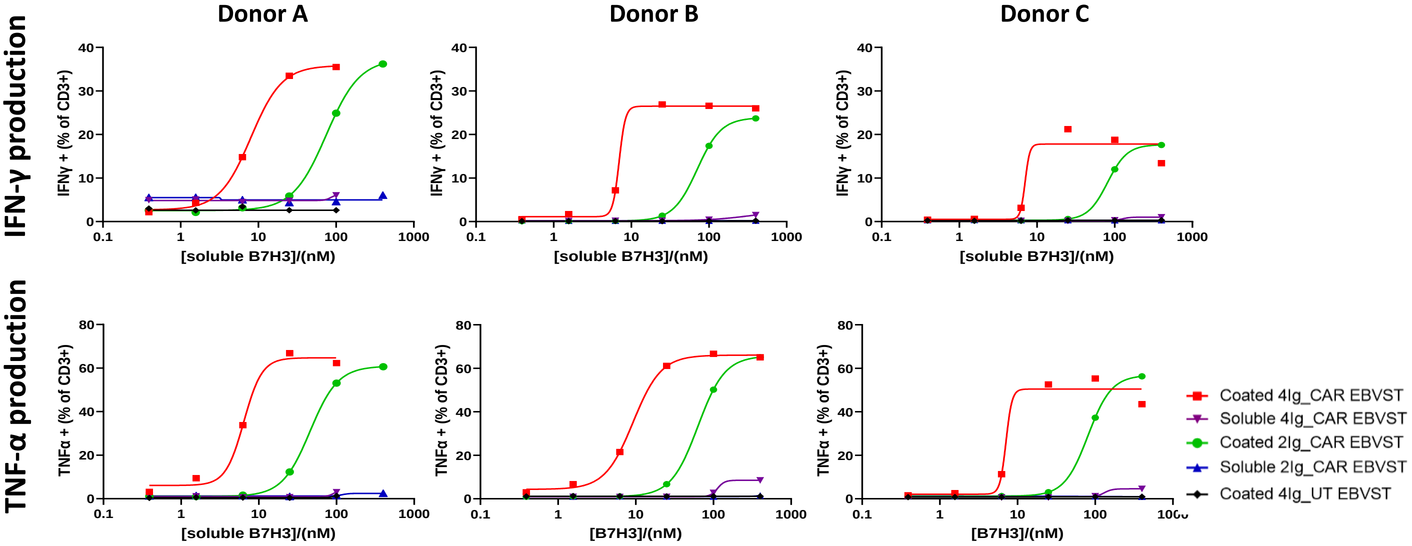

Supplementary figure 3. Further characterization of B7H3.CAR EBVSTs and their specificity for B7-H3. **A**, Schematic representation of the B7H3.CAR consisting of a B7-H3-targeting VHH clone P2A5, 4-1BB derived spacer, CD28 transmembrane domain, and CD28 and CD3ζ signalling domains (left panel). A truncated version of the B7H3.CAR (right panel) lacking functional signaling domains was generated as a control. **B,** Manufacturing protocol to generate CAR EBVSTs. **C,** CD4+ and CD8+ T cell frequencies among EBVSTs at final harvest. **D,** Memory subset proportions among EBVSTs at final harvest based on cell surface staining of CCR7 and CD45RA. Tem, effector memory; TEMRA, terminally differentiated effector memory expressing CD45RA; Tcm, central memory. **E,** Kinetics of B7H3.CAR EBVST cytolysis against B7-H3+ NCI-N87 gastric cancer cells and their B7-H3 knock-out counterparts at a 1:1 Effector : Target ratio. **F,** IFN-γ levels in supernatants after co-incubation of B7H3.CAR EBVSTs with N87 or N87-B7H3KO tumor cells. **G,** Cytotoxicity of ATCs expressing full length or truncated B7H3.CAR or no CAR against wild type MKN-45 gastric cancer cells. **H**, Percentages of CD3+ EBVSTs expressing IFNγ (top) or TNFα (bottom) after overnight stimulation with plate-coated or soluble B7-H3. Data presented are from 6 **(C and D)** or 3 **(E-H)** healthy donors. Error bars represent means ± SD where applicable.
