# Supplementary material for: B7-H3–Targeting Chimeric Antigen Receptors Epstein-Barr Virus–specific T Cells Provides a Tumor Agnostic Off-The-Shelf Therapy Against B7-H3–positive Solid Tumors
Source: Cancer Res Commun. 2024 Jun 4;4(6):1410–29. doi: 10.1158/2767-9764.CRC-23-0538 (PMC11149603; doi:10.1158/2767-9764.CRC-23-0538)
Supplement: Supplementary figure 4 [file crc-23-0538-s06.pdf]

Supplementary figure 4

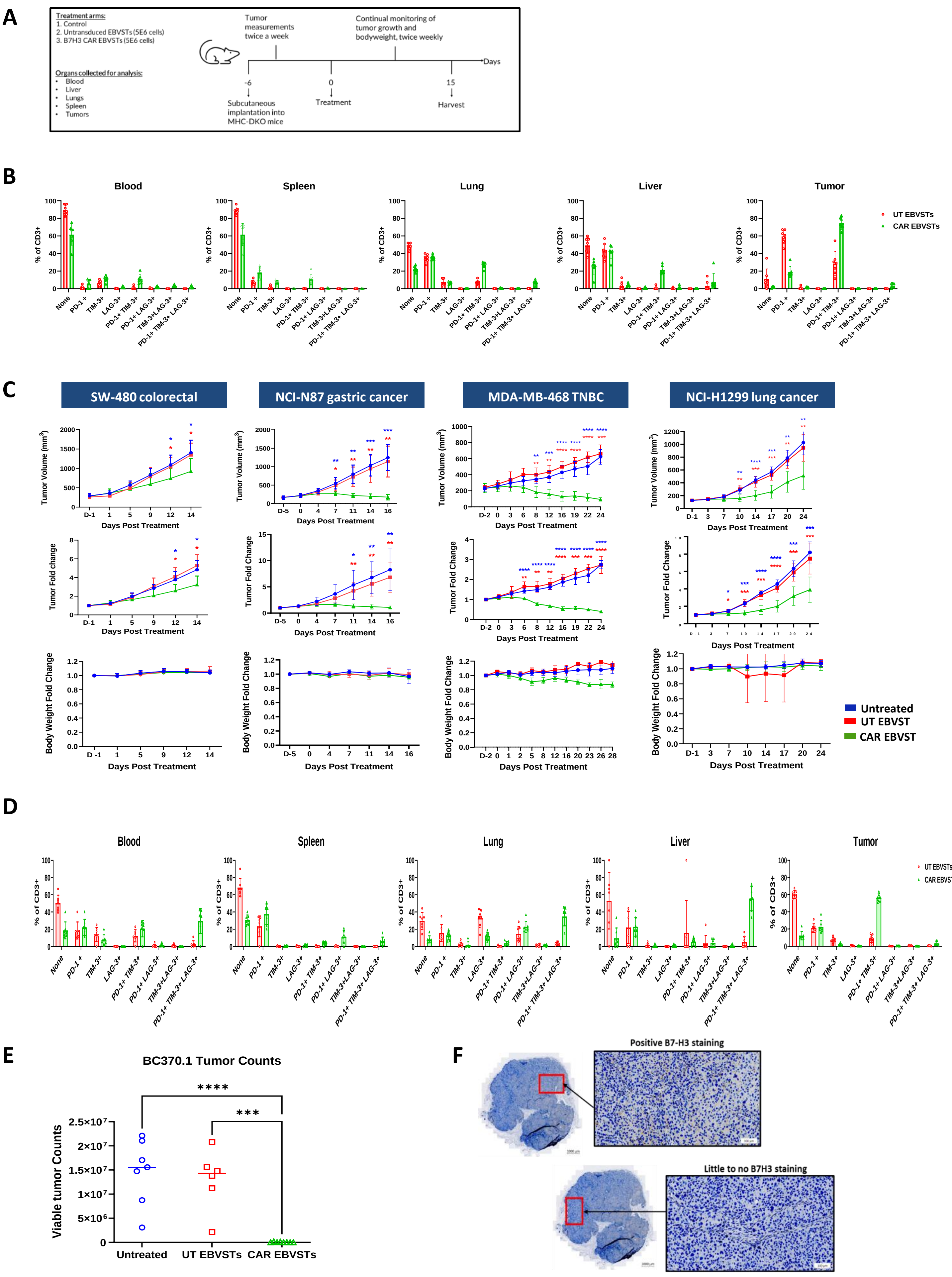

Supplementary figure 4

G

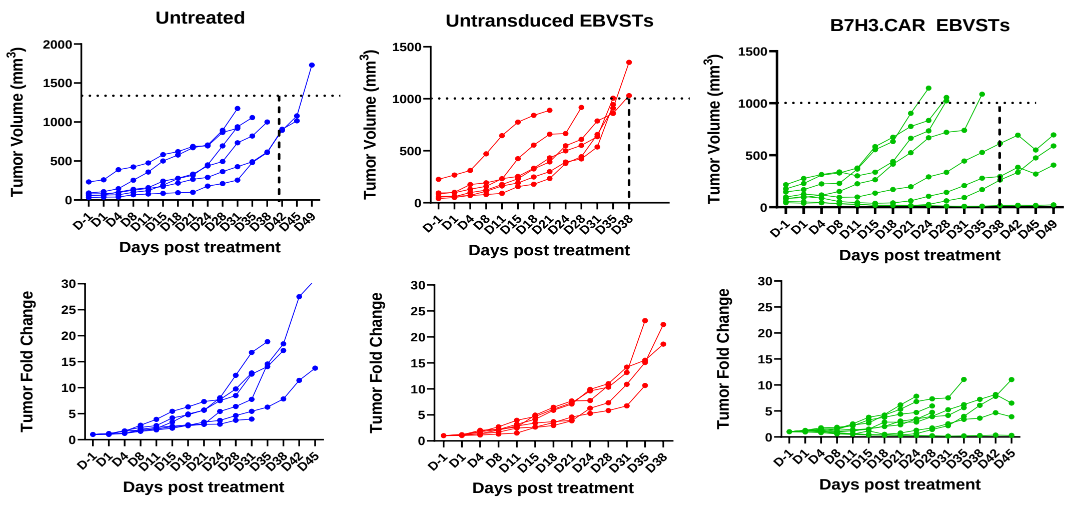

H

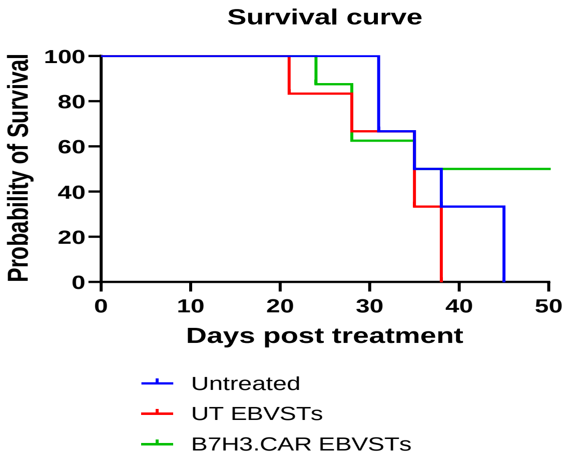

Supplementary figure 4. *In vivo* activities of B7H3.CAR EBVSTs against B7-H3 positive solid tumors. **A,** Experiment set-up to evaluate in vivo efficacy of B7H3.CAR EBVSTs in cell line-derived solid tumor models. **B,** Percentages of human CD3+ cells expressing the exhaustion markers in the blood, spleen, lungs, liver and tumor of mice in the HT-29 CRC model. **C,** Tumor volumes, fold change in tumor volumes, body weight measurements of untreated, UT and B7H3.CAR EBVST treatment groups in the SW-480, NCI-N87, MDA-MB-468 and NCI-H1299 cell-line derived solid tumor models. **D,** Percentages of human CD3+ cells expressing the exhaustion markers in the blood, spleen, lungs, liver and tumor of mice in the NCI-N87 model. **E,** Endpoint viable tumor counts of BC370.1 breast cancer PDX model. Each point on the graph represents a mouse. **F,** IHC staining of B7-H3 on lung cancer PDX (left) with representative magnified images (right). Brown color represents positive B7-H3 staining. Blue color represents cell nucleus. **G,** Tumor volumes and fold change in tumor volume measurements of lung cancer PDX. **H,** Kaplan-Meier log-rank survival curve.

Data comprises of 6-8 **(B-E, G-H)** mice in each treatment arm. For **C**, groups were compared using 2-way ANOVA with Tukey's test for multiple comparisons test. Comparison and p-values between untreated versus untransduced T, untreated versus B7H3.CAR EBVSTs and untransduced T versus B7H3.CAR EBVSTs groups is represented in black, blue and red, respectively. For **E**, groups were compared using 1-way ANOVA with Tukey's test for multiple comparisons test *: $p<0.05$; **: $p<0.01$, ***: $p<0.001$, ****: $p<0.0001$. Error bars denote means ± SD where applicable.
