# Supplementary material for: B7-H3–Targeting Chimeric Antigen Receptors Epstein-Barr Virus–specific T Cells Provides a Tumor Agnostic Off-The-Shelf Therapy Against B7-H3–positive Solid Tumors
Source: Cancer Res Commun. 2024 Jun 4;4(6):1410–29. doi: 10.1158/2767-9764.CRC-23-0538 (PMC11149603; doi:10.1158/2767-9764.CRC-23-0538)
Supplement: Supplementary figure 5 [file crc-23-0538-s07.pdf]

Supplementary figure 5

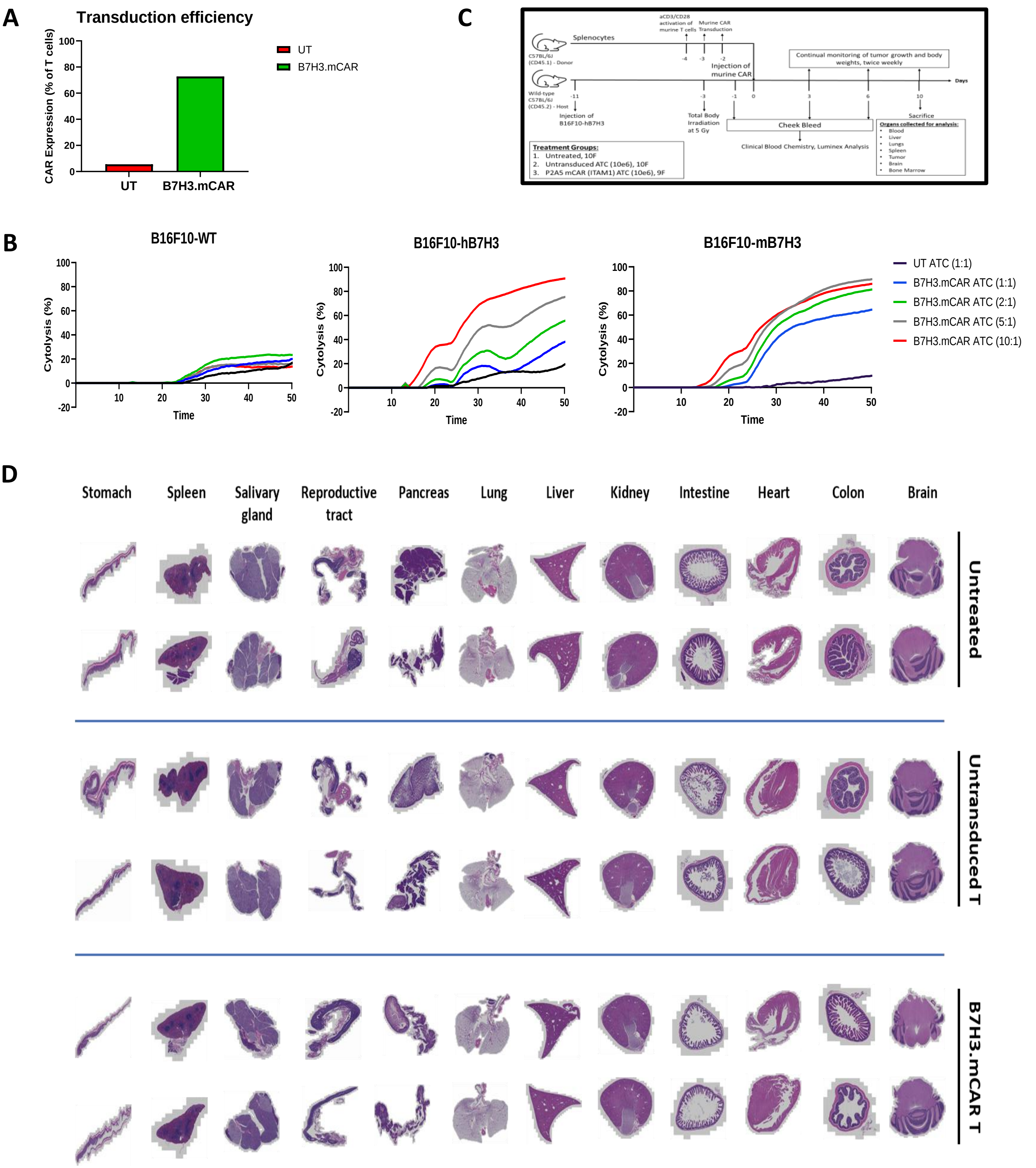

Supplementary figure 5. Generation and in-vitro efficacy of B7H3.mCAR T cells. **A,** Murine CAR expression in B7H3.mCAR T cells. **B,** Cytotoxicity of B7H3.mCAR against B16F10-WT, B16F10 engineered to express human B7-H3 (B16F10-hB7H3) and mouse B7-H3 (B16F10-mB7H3). **C**, Experimental scheme describing syngeneic mouse model to evaluate on-tumor off-target toxicities associated with B7H3 murine CAR T cells. **D,** H and E images of organs from immunocompetent mice with syngeneic tumors that were untreated, treated with un-transduced or B7H3.mCAR T cell. Organs were harvested from 2 mice in each treatment group and sent for pathology evaluation.
