# Supplementary material for: B7-H3–Targeting Chimeric Antigen Receptors Epstein-Barr Virus–specific T Cells Provides a Tumor Agnostic Off-The-Shelf Therapy Against B7-H3–positive Solid Tumors
Source: Cancer Res Commun. 2024 Jun 4;4(6):1410–29. doi: 10.1158/2767-9764.CRC-23-0538 (PMC11149603; doi:10.1158/2767-9764.CRC-23-0538)
Supplement: Supplementary figure 7 [file crc-23-0538-s09.pdf]

Supplementary figure 7

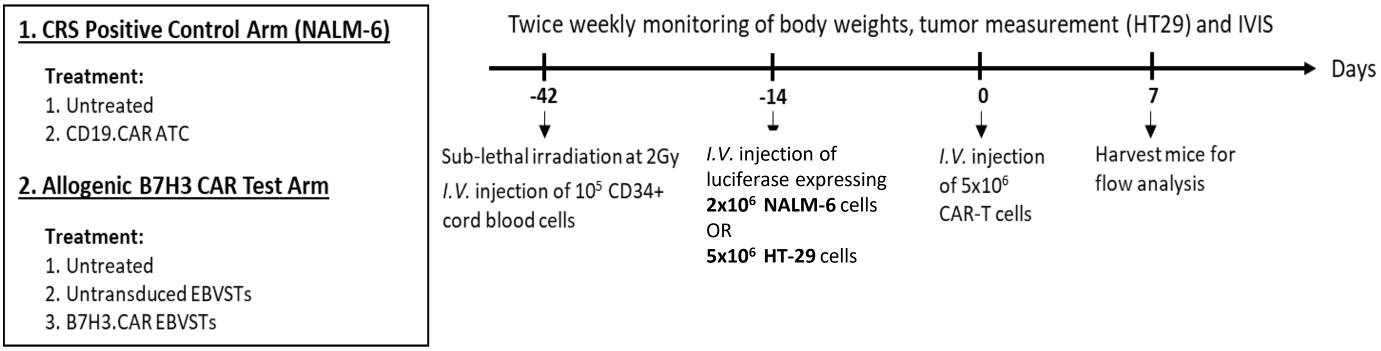

Supplementary figure 7. Schematic diagram of study set up to evaluate cytokine-release syndrome associated with allogeneic B7H3.CAR EBVSTs.
