# Supplementary material for: B7-H3–Targeting Chimeric Antigen Receptors Epstein-Barr Virus–specific T Cells Provides a Tumor Agnostic Off-The-Shelf Therapy Against B7-H3–positive Solid Tumors
Source: Cancer Res Commun. 2024 Jun 4;4(6):1410–29. doi: 10.1158/2767-9764.CRC-23-0538 (PMC11149603; doi:10.1158/2767-9764.CRC-23-0538)
Supplement: Supplementary figure 8 [file crc-23-0538-s10.pdf]

Supplementary figure 8

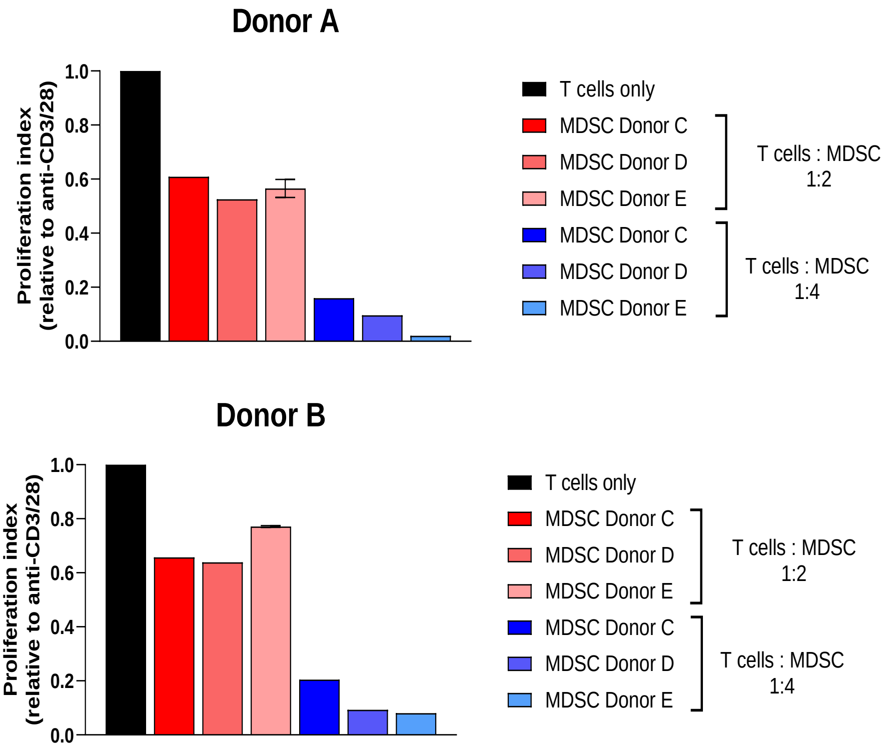

Supplementary figure 8. In vitro generated MDSC inhibits T cell proliferation. Proliferation index of anti-CD3/CD28 stimulated T cells from 2 independent donors, in the presence or absence of MDSCs generated from 3 other donors, at T cells: MDSC ratios of 1:2 and 1:4. Proliferation index was calculated by normalising percentages of proliferated cells against no MDSC control condition.
